# Sex differences in metabolically healthy and metabolically unhealthy obesity among Chinese children and adolescents

**DOI:** 10.3389/fendo.2022.980332

**Published:** 2022-10-14

**Authors:** Shan Cai, Jiajia Dang, Panliang Zhong, Ning Ma, Yunfei Liu, Di Shi, Zhiyong Zou, Yanhui Dong, Jun Ma, Yi Song

**Affiliations:** ^1^ Institute of Child and Adolescent Health, School of Public Health, Peking University, Beijing, China; ^2^ National Health Commission Key Laboratory of Reproductive Health, Peking University, Beijing, China

**Keywords:** obesity phenotypes, metabolically healthy obesity, metabolically unhealthy obesity, children and adolescents, sex differences

## Abstract

**Objectives:**

To analyze sex differences in the prevalence of obesity phenotypes and their risk factors among children and adolescents aged 7-18 years in China.

**Methods:**

We enrolled 15,114 children and adolescents aged 7-18 years into the final analysis. Obesity phenotypes were classified by body mass index (BMI) and metabolic status as metabolically healthy or unhealthy obesity. In addition, we collected four possible influencing factors on obesity phenotypes through questionnaires, including demographic, parental, early life, and lifestyle indicators. Multinomial logistic regression analysis in a generalized linear mixed model (GLMM) was selected to estimate the odds ratio (OR) and 95% confidence interval (95% CI) for identifying risk factors and control the cluster effects of schools. More importantly, the interaction terms of sex and each indicator were established to demonstrate the sex differences.

**Results:**

The prevalence of metabolically healthy obesity (MHO), metabolically unhealthy obesity (MUO), metabolically healthy overweight and obesity (MHOO), and metabolically unhealthy overweight and obesity (MUOO) were 3.5%, 5.6%, 11.1%, and 13.0% respectively, with higher prevalence in boys (5.3% vs. 1.6%, 7.9% vs. 3.1%, 14.3% vs. 7.7%, 15.6% vs. 10.1%). In addition, younger ages, single children, parental smoking, parental history of diseases (overweight, hypertension, diabetes), caesarean, premature, and delayed delivery time, high birth weight, insufficient sleep time, and excessive screen time were considered as important risk factors of MHO and MUO among children and adolescents (*p* < 0.05). More notably, boys were at higher risks of MUO when they were single children (boys: OR = 1.56, 95% CI: 1.24-1.96; girls: OR = 1.12, 95% CI: 0.82-1.54), while girls were more sensitive to MUO with parental smoking (girls: OR = 1.34, 95% CI: 1.02-1.76; boys: OR = 1.16, 95% CI: 0.97-1.39), premature delivery (girls: OR = 3.11, 95% CI: 1.59-6.07; boys: OR = 1.22, 95% CI: 0.67-2.22), high birth weight (girls: OR = 2.45, 95% CI: 1.63-3.69; boys: OR = 1.28, 95% CI: 0.96-1.70), and excessive screen time (girls: OR = 1.47, 95% CI: 1.06-2.04; boys: OR = 0.97, 95% CI: 0.79-1.20), with significant interaction term for sex difference (*p_interaction_* < 0.05).

**Conclusions:**

MHO and MUO are becoming prevalent among Chinese children and adolescents. Significant sex differences in the prevalence of obesity phenotypes as well as their environmental and genetic risk factors suggest it might be necessary to manage obesity phenotypes problems from a sex perspective.

## 1 Background

With rapid economic growth and changing lifestyles, being overweight and obese is becoming a global public health crisis (1, 2). China has the largest number of overweight and obese persons worldwide, with about 20% of children and adolescents suffering from one (3, 4). Childhood obesity is a major risk factor for non-communicable diseases including cardiovascular diseases, type 2 diabetes, musculoskeletal disorders, some cancers, and mental disorders, resulting in a higher chance of premature death and disability in adulthood (5, 6). What is more, further research found that the metabolically unhealthy group in the population with obesity had a higher risk of cardiovascular disease and all-cause mortality than the metabolically healthy group (7). Therefore, it is currently recommended to divide obesity into different phenotypes according to body mass index (BMI) and metabolic status, including metabolically healthy obesity (MHO) and metabolically unhealthy obesity (MUO), which should be given different interventions (7–10). However, there is no internationally agreed standard for the selection and cut-offs of metabolic indicators in the definition of obesity phenotypes, causing the estimated prevalence of MHO among children and adolescents to range from 7% to 21% (11–14). Because of this, Damanhoury et al. conducted a scope review to put forward the definition of obesity phenotypes based on international consensus in 2018, but it failed to reach an agreement on blood glucose as a metabolic indicator (15). Then, China issued an expert consensus to specify the definition of obesity phenotypes in 2019, including the measurement and cutoff of blood glucose (16). But till now, few studies have estimated the overall prevalence of MHO and MUO among children and adolescents under the above definition criteria, especially in China as a country with a heavy burden of obesity.

Identifying risk factors for obesity among children and adolescents is a significant way to prevent and control obesity. Previous studies generally believed that genetics and environmental factors all contributed to obesity (17–19). Consequently, the World Health Organization (WHO) specifically called for improvement of obesity among children and adolescents from parental behaviors, early life status, personal diets, and physical activity (20–22). Current research on the risk factors of obesity phenotypes focused on the comparative analysis of MHO and MUO (23–26), and the conclusions were not consistent between studies, especially for individual lifestyle factors such as sleep duration, screen time, physical activity, and so on (25, 26). Moreover, few studies have identified risk factors for MHO/MUO in the general population compared with a non-obesity group. Further, although numerous studies have shown that boys were at the greater risk of obesity (1, 27), as well as having higher abnormalities of cardiovascular risk factors (28, 29), there is little research evidence of sex differences in the prevalence of metabolically healthy and metabolically unhealthy obesity and their risk factors.

We hypothesized that the prevalence of obesity phenotypes were associated with demographic status, parental factors, early life status, as well as individual lifestyles, with sex differences possibly existing in both prevalence and risk factors. Consequently, the purpose of this study was to analyze the prevalence of obesity phenotypes and their risk factors according to the obesity phenotypes criteria based on international consensus in the total population of children and adolescents aged 7-18 years from a nationwide cross-sectional study in China in 2013, and additionally explore sex differences.

## 2 Methods

### 2.1 Study design and participants

The nationwide cross-sectional study was performed in September 2013, and selected children and adolescents aged 7-18 years from seven provinces using a multistage cluster random sampling method across mainland China, covering Tianjin, Shanghai, Hunan, Ningxia, Liaoning, Chongqing, and Guangdong. From this, 3-4 districts based on different economic levels from each province were randomly selected. Then, 12-16 schools were chosen from each district randomly. Finally, 2-3 classes per grade were selected randomly from each school. Further, based on previous medical history and examination, children and adolescents with severe conditions were excluded from the survey, including serious organ diseases, abnormal physical developments, and physical deformity. The detailed study design has been described carefully in a previous study (30). This study has been approved by the Ethical Committee of the Peking University (NO. IRB0000105213034), with all participants and their parents signing their informed consent voluntarily.

**Figure 1:**
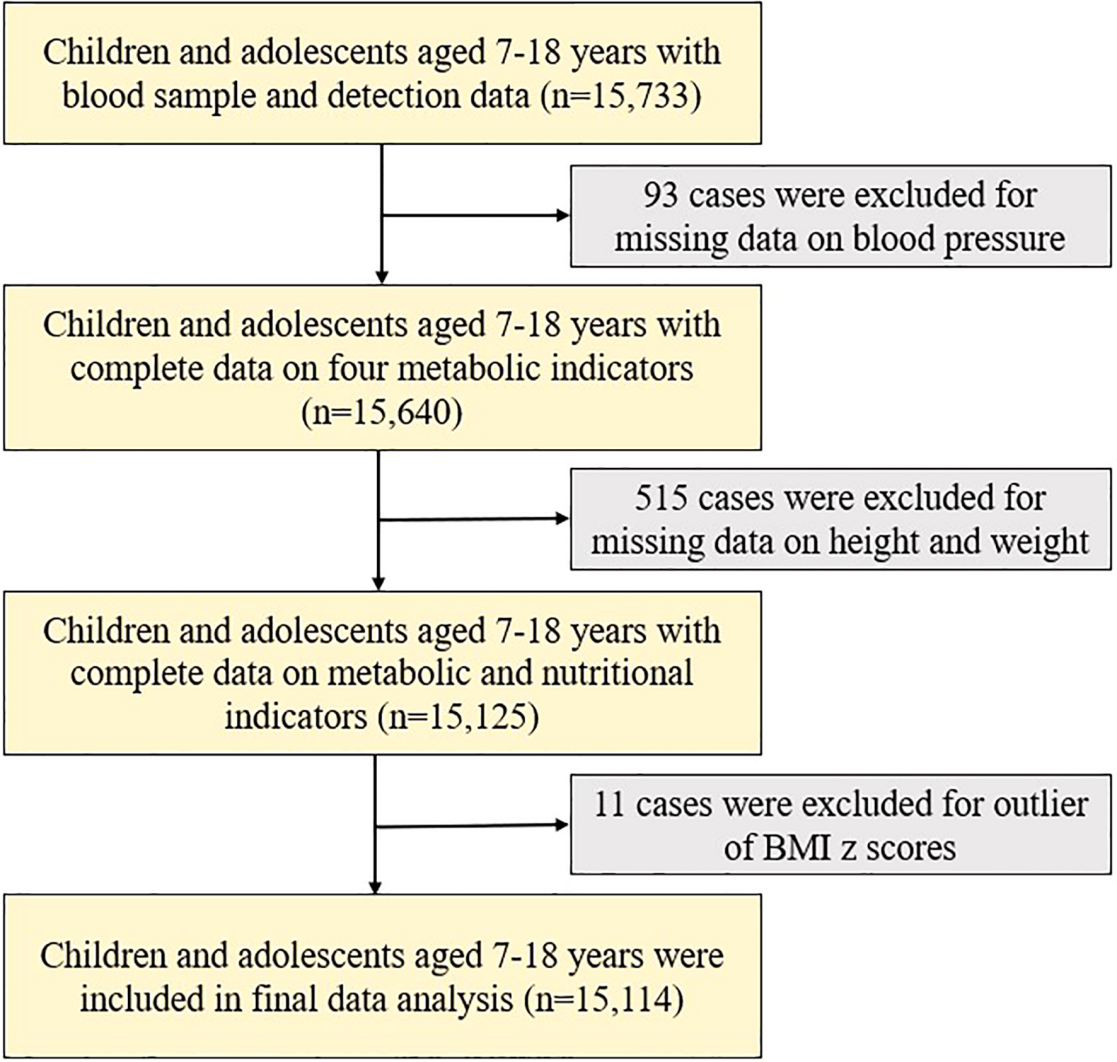
Flow chart of data. BMI, Body Mass Index.

According to the purpose of the study, we chose 15,733 children and adolescents aged 7-18 with complete blood sample as the primary sample of the study, among which we excluded cases lacking blood pressure data (n = 93), lacking height or weight information (n = 515), and those with BMI outliers defined as sex- and age-specific BMI Z scores < -5 or > 5 (n = 11). We enrolled 15,114 children and adolescents aged 7-18 years into the final analysis (**Figure 1**).

### 2.2 Data collection and questionnaire survey

#### 2.2.1 Anthropometric measurements

All anthropometric indicators were measured following a standardized procedure by a trained team. Height was measured by the portable stadiometer (model TZG, China) to the nearest 0.1 centimeter (cm), and weight was measured by the lever-type weight scale (model RGT-140, China) to the nearest 0.1 kilogram (kg), requiring participants to stand straight without shoes and wear light clothes. Blood pressure (BP), including systolic BP (SBP) and diastolic BP (DBP), were measured by a mercury sphygmomanometer (model XJ1ID, China) with participants sitting quietly for at least 5 min prior. All indicators were measured twice, based on which the average value was calculated for the final analysis. Significantly, about 5% of the participants would be rechecked per day, and all the participants would be measured again if the error exceeded 10%, ensuring the quality of measured data.

#### 2.2.2 Blood sample collection and detection

Firstly, venous blood was collected by venipuncture after fasting for 12 hours. Secondly, serum was separated by centrifuging at 3000 rpm for 10 min and transported to the experimental center at low temperature (−80 °C). Finally, a biomedical analysis company certified by Peking University conducted the blood biochemical analyses (30), using the hexokinase method, enzymatic method, and clearance method to measure fasting plasma glucose (FPG), triglyceride (TG), and high-density lipoprotein-cholesterol (HDL-C) respectively.

#### 2.2.3 Questionnaire survey

Information on the children and adolescents aged 7-18 years were collected using two questionnaires filled in by the participants and one of their parents respectively. The questionnaire filled in by children and adolescents was used to collect data on demographic characteristics (age, sex, residence, single-child status, and so on), lifestyle information including physical activity time, screen time, sleep duration, and the consumption of common food such as fruits, vegetables, and sugar-sweetened beverages, among which we collected the frequency (days) and amount (servings) of each food over a week. Then we calculated the average daily intake of a single food by the following equation: (days × quantity in each of those days)/7 (31). The parental questionnaire was used to obtain data on parental information (education level, smoking and drinking behaviors, history of obesity, hypertension, diabetes, and so on) and early life indicators (delivery model, delivery time, birth weight, and breastfeeding duration for children and adolescents). It was noteworthy that the questionnaires were revised and validated by experts in the early stage of our project. About 3% of questionnaires were re-examined and filled in by the same participants within a week. All questionnaires were also checked for logicality and integrity, making sure they were deemed feasible and acceptable for participants and their parents.

### 2.3 Definition and categorization of indicators

#### 2.3.1 Obesity phenotypes

Obesity phenotypes were classified according to nutrition and metabolic status as follows (15, 16): for the nutrition status, we used the WHO-recommended standard to calculate BMI-for-age Z scores, among which BMI was calculated as bodyweight (kg) divided by height squared (m^2^), and Z score was calculated as a child’s BMI minus the median BMI, divided by the standard deviation (SD) for that child’s age and sex in the WHO reference population (32). Then we divided nutritional status according to BMI Z scores in children and adolescents aged between 7-18 years for the following four outcomes: thinness (< -2 for BMI Z scores), normal weight (-2-1 for BMI Z scores), overweight (1-2 for BMI Z scores), and obesity (> 2 for BMI Z scores) (3). For metabolic status, metabolically unhealthy status was defined with 1-4 following metabolic abnormalities (15, 16) (1): SBP or DBP ≥ 90th percentile for sex- and age-specific group (33) (2); FPG ≥ 5.6 mmol/L (3); TG ≥ 1.70 mmol/L; and (4) HDL-C < 1.03 mmol/L. Metabolically healthy normal weight (MHNW) was defined as normal weight with metabolically healthy status. Metabolically unhealthy normal weight (MUNW) was defined as normal weight with metabolically unhealthy status. Metabolically healthy overweight (MHOW) was defined as overweight with metabolically healthy status. Metabolically unhealthy overweight (MUOW) was defined as overweight with metabolically unhealthy status. MHO was defined as obesity with metabolically healthy status. MUO was defined as obesity with metabolically unhealthy status. Metabolically healthy overweight and obesity (MHOO) was defined as overweight and obese with metabolically healthy status. Metabolically unhealthy overweight and obesity (MUOO) was defined as overweight and obese with metabolically unhealthy status.

#### 2.3.2 Demographic indicators

In this study, age, sex, residence, single-child status, and parental education level were considered as possible demographic influencing factors for obesity phenotypes among children and adolescents. Parental education level was divided into junior high school and below and senior high school and above, with the highest education level of parents (either mother or father) also considered (34).

#### 2.3.3 Parental indicators

According to the recommendations in China on smoking and drinking for adults, we divided smoking status into “Yes/no”, and defined drinking status as “moderate (≤ 25g alcohol for men and ≤ 15g alcohol for women per day)” and “excessive (> 25g alcohol for men and > 15g alcohol for women per day)” (35). Overweight (including obesity) in adulthood was defined as BMI ≥ 25 kg/m^2^ (1). For smoking, drinking excessively, overweight, hypertension, and diabetes, as long as either parent was positive for one category, it was considered that the parents had the above situation.

#### 2.3.4 Early life indicators

Delivery model, delivery time, birth weight, and breastfeeding duration were selected as possible early life factors influencing obesity phenotypes among children and adolescents. Delivery model included eutocia and caesarean. Delivery time was categorized into premature (prior to 37 weeks gestation), delayed (latter to 42 weeks gestation), and normal delivery time (36). Birth weight was divided into low birth weight (LBW, birth weight < 2500g), normal birth weight (NBW, birth weight: 2500-4000g), and high birth weight (HBW, birth weight ≥ 4000g) (37). Breastfeeding duration was separated into 0-5 months and ≥ 6 months (22), for there were too few non-breastfed and 1-5 months breastfed children and adolescents.

#### 2.3.5 Lifestyle indicators

Dietary consumption, physical activity, screen time, and sleep duration were common risk factors of obesity as mentioned. According to the dietary guidelines for school-age children in China (2022), children and adolescents aged 7-18 years should eat at least 150g fruits and 300 vegetables per day, and weekly intake ≤ 250 mL sugar-sweetened beverage (38). Children and adolescents should also maintain at least 1 hour of moderate to vigorous physical activity and 9 hours of sleep duration, with no more than 2 hours of screen time (31). We divided each lifestyle into two categories according to the above cut-offs.

### 2.4 Statistical analysis

Chi-squared test was used to compare basic characteristic differences between boys and girls; it was also performed to examine the differences between primary sample and final sample, showing no statistical changes in age, sex, and residence (*p* > 0.05).

The association between obesity phenotypes and risk factors was determined by the multinomial logistic regression analysis in a generalized linear mixed model (GLMM) to control the cluster effects of schools, taking the individual participants as the fixed-effect term and the school address as the random-effect term. In this study, we have mainly established four regression models for analysis. Model 1 was performed to research the risk factors of MHO and MUO, taking the participants without MHO or MUO as the reference group. Model 2 was performed to analyze the risk factors of MHOO and MUOO, taking the participants without MHOO or MUOO as the reference group. Model 3 and Model 4 were sensitivity analysis, replacing the original reference group of Model 1 and Model 2 with MHNW respectively. In addition, we conducted a sex-specific comparative analysis of the prevalence of obesity phenotypes at each indicator subgroup level to identify sex differences. Moreover, the interaction terms of sex and each indicator were also tested in model 1 and model 2 to demonstrate sex differences in association between obesity phenotypes and risk factors.

A two-tailed *p-value* < 0.05 was considered statistically significant in the whole process. All statistical analyses were performed with IBM SPSS Statistics version 26.0 and R 4.0.5.

## 3 Results

### 3.1 Characteristics of study participants

A total of 15,114 children and adolescents were analyzed in the study and the characteristics of the sample by sex are shown in **Table 1**. For demographic indicators, there were significant differences in the distribution of age groups between boys and girls (*p* < 0.05). The proportions of single-child status and the education level of junior high school and below in parents were higher in boys (*p* < 0.05). For parental indicators, parental smoking, being overweight, and having hypertension were in higher proportion in girls (*p* < 0.05). For early life indicators, delivery model and birth weight were unevenly distributed among boys and girls (*p* < 0.05). For lifestyle indicators, girls were more likely to take in ≥ 150g/d fruits and ≤ 250ml/w beverage, maintain ≤ 2h/d screen time, while boys were more likely to take at least 1 hour of moderate to vigorous physical activity (*p* < 0.05). Importantly, nutritional status differed between boys and girls, where the prevalence of obesity in boys was higher than that in girls (13.2% vs. 4.8%, *p* < 0.05). Moreover, boys had higher proportion for abnormal FPG and HDL-C (*p* < 0.05), and girls had higher proportion for abnormal TG (*p* < 0.05), while clustered metabolic status failed to reach significance between boys and girls (*p* > 0.05). Most importantly, there were statistical differences in nutritional and metabolic status among boys and girls (*p* < 0.001), where the prevalence of MHO, MUO, MHOO, MUOO were 5.3%, 7.9%, 14.3%, 15.6% respectively in boys, and 1.6%, 3.1%, 7.7%, 10.1% in girls respectively.

**Table 1:** Characteristics of the study participants.

	Boys (N = 7,750)	Girls (N = 7,364)	Total (N = 15,114)	*P-value*
**Demographic indicators**				
Age (years)				0.003
7-9	2702 (34.9)	2578 (35.0)	5280 (34.9)	
10-12	2101 (27.1)	1829 (24.8)	3930 (26.0)	
13-18	2947 (38.0)	2957 (40.2)	5904 (39.1)	
Residence				0.217
Rural	2930 (37.8)	2856 (38.8)	5786 (38.3)	
Urban	4820 (62.2)	4508 (61.2)	9328 (61.7)	
Single-child status				< 0.001
Yes	5825 (75.2)	4942 (67.1)	10767 (71.2)	
No	1925 (24.8)	2422 (32.9)	4347 (28.8)	
Parental education level				< 0.001
Senior high school and above	3903 (50.4)	3974 (54.0)	7877 (52.1)	
Junior high school and below	3847 (49.6)	3390 (46.0)	7237 (47.9)	
**Parental indicators**				
Parental Smoking				0.004
Yes	3234 (41.7)	3242 (44.0)	6476 (42.8)	
No	4516 (58.3)	4122 (56.0)	8638 (57.2)	
Parental drinking				0.824
Excessive	182 (2.3)	177 (2.4)	359 (2.4)	
Moderate	7568 (97.7)	7187 (97.6)	14755 (97.6)	
Parental overweight				0.035
Yes	2763 (35.7)	2747 (37.3)	5510 (36.5)	
No	4987 (64.3)	4617 (62.7)	9604 (63.5)	
Parental hypertension				0.008
Yes	428 (5.5)	482 (6.5)	910 (6.0)	
No	7322 (94.5)	6882 (93.5)	14204 (94.0)	
Parental diabetes				0.102
Yes	131 (1.7)	151 (2.1)	282 (1.9)	
No	7619 (98.3)	7213 (97.9)	14832 (98.1)	
**Early life indicators**				
Delivery model				< 0.001
Caesarean	4175 (53.9)	3662 (49.7)	7837 (51.9)	
Eutocia	3575 (46.1)	3702 (50.3)	7277 (48.1)	
Delivery time				0.842
Premature delivery	143 (1.8)	140 (1.9)	283 (1.9)	
Delayed delivery	114 (1.5)	116 (1.6)	230 (1.5)	
Normal	7493 (96.7)	7108 (96.5)	14601 (96.6)	
Birthweight				< 0.001
HBW	675 (8.7)	430 (5.8)	1105 (7.3)	
LBW	229 (3.0)	269 (3.7)	498 (3.3)	
NBW	6846 (88.3)	6665 (90.5)	13511 (89.4)	
Breastfeeding				0.239
0-5m	1721 (22.2)	1577 (21.4)	3298 (21.8)	
≥6m	6029 (77.8)	5787 (78.6)	11816 (78.2)	
**Lifestyle indicators**				
Fruits				0.048
<150g/d	5690 (73.4)	5301 (72.0)	10991 (72.7)	
≥150g/d	2060 (26.6)	2063 (28.0)	4123 (27.3)	
Vegetables				0.701
<300g/d	6357 (82.0)	6058 (82.3)	12415 (82.1)	
≥300g/d	1393 (18.0)	1306 (17.7)	2699 (17.9)	
Beverage				< 0.001
>250ml/w	4695 (60.6)	3628 (49.3)	8323 (55.1)	
≤250ml/w	3055 (39.4)	3736 (50.7)	6791 (44.9)	
Sleep time				0.426
<9h/d	6370 (82.2)	6089 (82.7)	12459 (82.4)	
≥9h/d	1380 (17.8)	1275 (17.3)	2655 (17.6)	
Screen time				< 0.001
>2h/d	1816 (23.4)	1333 (18.1)	3149 (20.8)	
≤2h/d	5934 (76.6)	6031 (81.9)	11965 (79.2)	
PA time				< 0.001
<1h/d	5254 (67.8)	5643 (76.6)	10897 (72.1)	
≥1h/d	2496 (32.2)	1721 (23.4)	4217 (27.9)	
**Nutritional status**				< 0.001
Obesity	1021 (13.2)	350 (4.8)	1371 (9.1)	
Overweight	1294 (16.7)	962 (13.1)	2256 (14.9)	
Normal weight	5157 (66.5)	5813 (78.9)	10970 (72.6)	
Thinness	278 (3.6)	239 (3.2)	517 (3.4)	
**Metabolic indicators**				
BP				0.633
Normal	6074 (78.4)	5795 (78.7)	11869 (78.5)	
Abnormal	1676 (21.6)	1569 (21.3)	3245 (21.5)	
FPG				< 0.001
Normal	7556 (97.5)	7286 (98.9)	14842 (98.2)	
Abnormal	194 (2.5)	78 (1.1)	272 (1.8)	
TG				0.001
Normal	6573 (84.8)	6102 (82.9)	12675 (83.9)	
Abnormal	1177 (15.2)	1262 (17.1)	2439 (16.1)	
HDL-C				< 0.001
Normal	6694 (86.4)	6562 (89.1)	13256 (87.7)	
Abnormal	1056 (13.6)	802 (10.9)	1858 (12.3)	
Metabolic status				0.113
Metabolically healthy	4673 (60.3)	4533 (61.6)	9206 (60.9)	
Metabolically unhealthy	3077 (39.7)	2831 (38.4)	5908 (39.1)	
**Nutritional and metabolic status**				< 0.001
MHO	408 (5.3)	119 (1.6)	527 (3.5)	
MUO	613 (7.9)	231 (3.1)	844 (5.6)	
MHOW	699 (9.0)	446 (6.1)	1145 (7.6)	
MUOW	595 (7.7)	516 (7.0)	1111 (7.4)	
MHNW	3360 (43.4)	3786 (51.4)	7146 (47.3)	
MUNW	1797 (23.3)	2027 (27.5)	3824 (25.3)	
Thinness	278 (3.6)	239 (3.3)	517 (3.5)	

P-value was regarding comparison between boys and girls; NBW, normal birth weight; LBW, low birth weight; LBW, high birth weight; PA, physical activity; BP, blood pressure; FPG, fasting plasma glucose; TG, triglyceride; HDL-C, high density lipoprotein-cholesterol; MHO, metabolically healthy obesity; MUO, metabolically unhealthy obesity; MHOW, metabolically healthy overweight; MUOW, metabolically unhealthy overweight; MHNW, metabolically healthy normal weight; MUNW, metabolically unhealthy normal weight.

### 3.2 The prevalence of obesity phenotypes and sex differences

The prevalence of obesity phenotypes in each indicator subgroup is shown in **Table 2**. Among different age groups in the total population, the highest prevalence of MHO was in 7-9 years group (6.3%), while the lowest was in 13-18 years group (1.1%). The prevalence of MHO and MUO were 3.9% and 5.0% in urban children and adolescents, respectively, with 2.9% and 6.5% in rural areas. In addition, children and adolescents who had single-child status, parental overweight, high birth weight, and caesarean model seemed in higher prevalence of MHO and MUO, especially for the parental overweight subgroup, which was almost as high as 2 times than that in the parental non-overweight subgroup. However, there appeared to be little differences in prevalence of obesity phenotypes among each lifestyle subgroups. It was worth noting that the prevalence of all levels of obesity phenotypes in boys was statistically higher than that in girls in the most subgroups (*p* < 0.05), except for some subgroups at some indicator levels including parental drinking, parental diabetes, delivery time, and birth weight.

**Table 2:** The prevalence of obesity phenotypes by sex.

	Boys (N = 7,750)				Girls (N = 7,364)				Total (N = 15,114)			
	MHO	MUO	MHOO	MUOO	MHO	MUO	MHOO	MUOO	MHO	MUO	MHOO	MUOO
***Demographic indicators***												
Age (years)												
7-9	9.2	7.5	20.6	12.4	3.2	3.1	10.9	9.2	6.3^#^	5.4^#^	15.9^#^	10.8^#^
10-12	5.0	10.0	15.7	18.9	1.4	3.9	7.8	12.8	3.3^#^	7.2^#^	12.0^#^	16.1^#^
13-18	1.8	6.7	7.5	16.1	0.4	2.7	4.8	9.3	1.1^#^	4.7^#^	6.1^#^	12.7^#^
Residence												
Rural	4.3	9.0	13.3	16.8	1.4	4.0	7.0	11.7	2.9^#^	6.5^#^	10.2^#^	14.3^#^
Urban	5.9	7.3	14.9	14.8	1.7	2.6	8.1	9.2	3.9^#^	5.0^#^	11.6^#^	12.1^#^
Single-child status												
Yes	5.4	8.5	14.7	16.5	1.9	3.1	8.2	9.9	3.8^#^	6.0^#^	11.7^#^	13.5^#^
No	4.9	6.1	13.1	12.7	0.9	3.2	6.7	10.6	2.7^#^	4.5^#^	9.5^#^	11.5^#^
Parental education level												
Senior high school and above	6.5	8.7	18.0	16.6	2.1	3.3	9.4	10.6	4.3^#^	6.0^#^	13.6^#^	13.6^#^
Junior high school and below	4.0	7.1	10.6	14.6	1.0	2.9	5.7	9.6	2.6^#^	5.2^#^	8.3^#^	12.2^#^
***Parental indicators***												
Parental Smoking												
Yes	5.8	9.0	15.2	16.5	1.7	3.9	8.7	12.0	3.8^#^	6.5^#^	11.9^#^	14.3^#^
No	4.8	7.1	13.6	14.9	1.6	2.5	6.9	8.7	3.3^#^	4.9^#^	10.4^#^	11.9^#^
Parental drinking												
Excessive	9.3	5.5	19.8	13.7	3.4	2.3	10.7	12.4	6.4*	3.9	15.3*	13.1
Moderate	5.2	8.0	14.2	15.6	1.6	3.2	7.6	10.1	3.4^#^	5.6^#^	11.0^#^	12.9^#^
Parental overweight												
Yes	7.9	12.1	19.3	21.5	2.7	5.4	11.6	14.5	5.3^#^	8.7^#^	15.4^#^	18.0^#^
No	3.8	5.6	11.5	12.3	1.0	1.8	5.3	7.5	2.5^#^	3.8^#^	8.6^#^	10.0^#^
Parental hypertension												
Yes	6.8	12.4	14.3	22.9	2.3	3.9	10.8	13.7	4.4^#^	7.9^#^	12.4*	18.0^#^
No	5.2	7.6	14.3	15.2	1.6	3.1	7.5	9.9	3.4^#^	5.4^#^	11.0^#^	12.6^#^
Parental diabetes												
Yes	9.2	12.2	20.6	19.8	2.6	7.9	13.9	15.2	5.7*	9.9	17.0	17.4
No	5.2	7.8	14.2	15.5	1.6	3.0	7.5	10.0	3.4^#^	5.5^#^	10.9^#^	12.9^#^
***Early life indicators***												
Delivery model												
Caesarean	6.5	8.6	16.0	16.0	2.2	3.6	9.0	10.2	4.5^#^	6.3^#^	12.7^#^	13.3^#^
Eutocia	3.8	7.0	12.3	15.1	1.0	2.7	6.4	10.0	2.4^#^	4.8^#^	9.3^#^	12.5^#^
Delivery time												
Premature delivery	7.7	9.8	17.5	14.7	0.7	8.6	6.4	15.7	4.2*	9.2	12.0^#^	15.2
Delayed delivery	1.8	8.8	13.2	15.8	3.4	5.2	7.8	17.2	2.6	7.0	10.4	16.5
Normal	5.3	7.9	14.2	15.6	1.6	3.0	7.7	9.9	3.5^#^	5.5^#^	11.1^#^	12.8^#^
Birthweight												
HBW	10.5	9.5	21.5	15.1	4.0	7.4	13.0	14.9	8.0^#^	8.7	18.2^#^	15.0
LBW	3.9	8.7	11.4	18.3	1.1	4.1	6.3	8.2	2.4	6.2*	8.6*	12.9^#^
NBW	4.8	7.7	13.7	15.5	1.5	2.8	7.4	9.9	3.2^#^	5.3^#^	10.6^#^	12.8^#^
Breastfeeding												
0-5m	6.6	7.3	17.0	14.2	2.4	2.5	10.5	8.2	4.6^#^	5.0^#^	13.9^#^	11.3^#^
≥6m	4.9	8.1	13.5	16.0	1.4	3.3	6.9	10.7	3.2^#^	5.7^#^	10.3^#^	13.4^#^
***Lifestyle indicators***												
Fruits												
<150g/d	5.1	7.4	13.7	15.1	1.4	2.8	7.2	9.7	3.3^#^	5.2^#^	10.6^#^	12.5^#^
≥150g/d	5.8	9.4	16.0	16.9	2.1	4.0	8.9	11.3	4.0^#^	6.7^#^	12.4^#^	14.1^#^
Vegetables												
<300g/d	5.1	7.7	13.9	15.3	1.5	3.0	7.4	10.1	3.4^#^	5.4^#^	10.8^#^	12.7^#^
≥300g/d	6.0	8.7	15.9	17.0	2.0	3.9	8.9	10.5	4.0^#^	6.4^#^	12.5^#^	13.9^#^
Beverage												
>250ml/w	4.9	7.6	13.2	16.3	1.4	3.0	7.6	10.0	3.4^#^	5.6^#^	10.8^#^	13.5^#^
≤250ml/w	5.8	8.3	15.9	14.6	1.8	3.3	7.8	10.3	3.6^#^	5.5^#^	11.4^#^	12.2^#^
Sleep time												
<9h/d	5.0	8.0	13.5	16.2	1.5	3.1	7.3	10.4	3.3^#^	5.6^#^	10.5^#^	13.3^#^
≥9h/d	6.7	7.7	17.8	12.9	2.4	3.4	9.5	8.9	4.6^#^	5.6^#^	13.8^#^	11.0^#^
Screen time												
>2h/d	5.8	7.3	15.5	13.9	1.9	4.1	9.0	10.7	4.1^#^	5.9^#^	12.7^#^	12.5^#^
≤2h/d	5.1	8.1	13.9	16.1	1.6	2.9	7.4	10.0	3.3^#^	5.5^#^	10.6^#^	13.0^#^
PA time												
<1h/d	5.3	7.4	14.1	15.0	1.6	3.1	7.6	10.0	3.4^#^	5.2^#^	10.7^#^	12.4^#^
≥1h/d	5.1	9.0	14.6	16.9	1.6	3.4	8.0	10.6	3.7^#^	6.7^#^	11.9^#^	14.3^#^

*Significant difference between boys and girls (p < 0.05); ^#^Significant difference between boys and girls (p < 0.01). NBW, normal birth weight; LBW, low birth weight; HBW, high birth weight; PA, physical activity; MHO, metabolically healthy obesity; MUO, metabolically unhealthy obesity; MHOO, metabolically healthy overweight and obesity; MUOO, metabolically unhealthy overweight and obesity.

### 3.3 Association between obesity phenotypes and risk factors by sex

**Figure 2:**
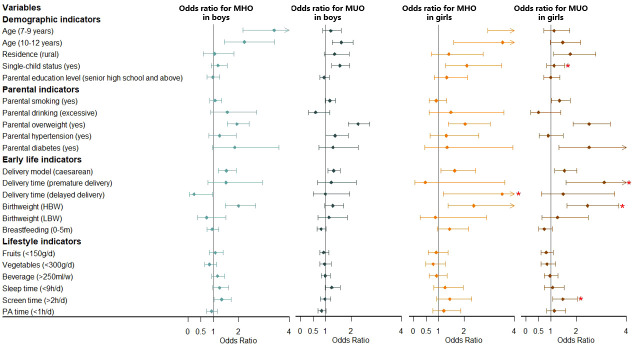
Association between MHO/MUO prevalence and risk factors stratified by sex. Obesity phenotypes include metabolically healthy obesity (MHO) and metabolically unhealthy obesity (MUO), taking the participants without MHO or MUO as the reference group. Adjusted for age, residence, single-child status, parental education level, parental smoking, parental drinking, parental overweight, parental hypertension, parental diabetes, delivery model, delivery time, birth weight, breastfeeding, fruits, vegetables, beverage, sleep time, screen time, physical activity (PA) time. *Significant interaction with sex in the whole model. NBW, normal birth weight; LBW, low birth weight; LBW, high birth weight.

**Figure 3:**
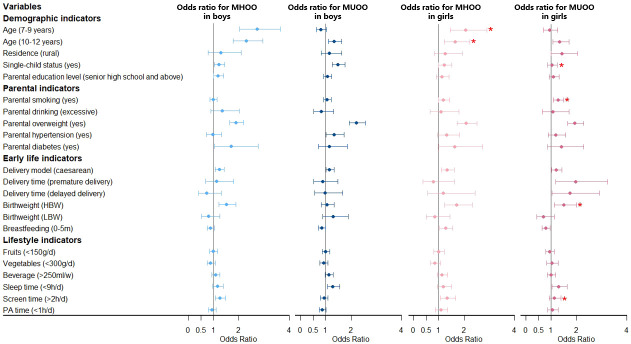
Association between MHOO/MUOO prevalence and risk factors stratified by sex. Obesity phenotypes include metabolically healthy overweight and obesity (MHOO) and metabolically unhealthy overweight and obesity (MUOO), taking the participants without MHOO or MUOO as the reference group. Adjusted for age, residence, single-child status, parental education level, parental smoking, parental drinking, parental overweight, parental hypertension, parental diabetes, delivery model, delivery time, birth weight, breastfeeding, fruits, vegetables, beverage, sleep time, screen time, physical activity (PA) time. *Significant interaction with sex in the whole model. NBW, normal birth weight; LBW, low birth weight; HBW, high birth weight.

Univariate analysis for risk factors for obesity phenotypes are shown in **Table S1** and **Table S2**, and multivariate analysis for model 1 and model 2 are shown in **Figure 2** (**Table S3**) and **Figure 3** (**Table S4**), with sensitive analysis shown in **Table S5** and **Table S6**, respectively. Univariate analysis for different metabolic states within each sex group were shown in **Table S7** and **Table S8**, and results about different metabolic states in overweight versus obese groups are in **Table S9**.

#### 3.3.1 Demographic indicators

Multivariate analysis for model 1 about risk factors for MHO and MUO is shown in **Figure 2** and **Table S3**. For boys, the 7-9 years group and 10-12 years group had a higher risk of MHO (OR = 3.40, 95% CI: 2.16-5.35; OR = 2.22, 95% CI: 1.44-3.42), with the 10-12 years group having a higher risk of MUO (OR = 1.62, 95% CI: 1.25-2.09) additionally, all compared with the 13-18 years group. Boys in single-child families were associated with a higher prevalence of MUO (OR = 1.56, 95% CI: 1.24-1.95). Model 2 (**Figure 3** and **Table S4**) showed similar results to model 1 (**Figure 2** and **Table S3**) mentioned above, analyzing the risk factors for MHOO and MUOO. Sensitivity analyses (**Table S5** and **Table S6**) were also similar except for the risk of MUO and MUOO at different age group levels, specifically observing a lower risk of MUOO (OR = 0.50, 95% CI: 0.37-0.68) in the 7-9 years group. For girls, higher risks of MHO (OR = 6.41, 95% CI: 2.94-13.95; OR = 3.53, 95% CI: 1.60-7.78) were discovered at the 7-9 years and 10-12 years groups. Apart from this, the prevalence of MUO (OR = 1.76, 95% CI: 1.11-2.78) was higher in rural residence and MHO (OR = 2.11, 95% CI: 1.27-3.49) was higher in single-child households. Model 2 (**Figure 3** and **Table S4**) showed similar results as model 1 (**Figure 2** and **Table S3**) Sensitivity analyses (**Table S5** and **Table S6**) were different in age and residence, showing the 7-9 years group was associated with a lower risk of MUOO (OR = 0.57, 95% CI: 0.40-0.82), with no statistical significance in residence.

Moreover, interaction tests showed statistical significance between sex with age and single-child status (*p_interaction_* < 0.05), respectively, indicating boys of 7-9 years and 10-12 years had a stronger association with higher prevalence of MHOO, and the same was for risks of MUO and MUOO in single-child status.

#### 3.3.2 Parental indicators

For boys, it was clearly observed that parental overweight was associated with a higher risk of MHO (OR = 1.93, 95% CI: 1.55-2.40) and MUO (OR = 2.28, 95% CI: 1.90-2.73) in model 1 (**Figure 2** and **Table S3**), with similar results in model 2 about MHOO and MUOO (**Figure 3** and **Table S4**). In addition, model 2 (**Figure 3** and **Table S4**) found that parental hypertensions were associated with higher MUOO (OR = 1.33, 95% CI: 1.02-1.73), and parental diabetes were associated with higher MHOO (OR = 1.70, 95% CI: 1.05-2.76). For girls, it was also obviously observed that parental overweight was associated with higher risks of MHO (OR = 2.04, 95% CI: 1.38-3.03) and MUO (OR = 2.51, 95% CI: 1.88-3.35), and parental smoking was associated with higher prevalence of MUO (OR = 1.34, 95% CI: 1.02-1.76), which were observed in model 1 (**Figure 2** and **Table S3**), with the same results in model 2 (**Figure 3** and **Table S4**) for MUOO. Parental diabetes was discovered to be associated with higher risk of MUO (OR = 2.51, 95% CI: 1.31-4.80) only. The above was the same for the sensitive analysis (**Table S5** and **Table S6**).

Furthermore, interaction tests showed girls were at higher risk of MUOO than boys in families with smoking parents (*p_interaction_* < 0.05).

#### 3.3.3 Early life indicators

Model 1 (**Figure 2** and **Table S3**) showed that boys who were born through caesarean in early life had higher risks of MHO (OR = 1.51, 95% CI: 1.20-1.90) and MUO (OR = 1.31, 95% CI: 1.09-1.57), while it was also in higher prevalence of MHO (OR = 1.99, 95% CI: 1.48-2.67) in high birth weight status. However, model 2 (**Figure 3** and **Table S4**) additionally found out the risk of MUOO (OR = 0.84, 95% CI: 0.71-0.99) in 0-5 months of breastfeeding was lower than that in ≥ 6 months of breastfeeding. As for girls, it was similarly in model 1 (**Figure 2** and **Table S3**) that being born through caesarean in early life had higher risks of MHO (OR = 1.63, 95% CI: 1.08-2.46) and MUO (OR = 1.53, 95% CI: 1.15-2.03), while it was observed in high birth weight status likewise with OR values were 2.39 (95% CI: 1.36-4.18), and 2.45 (95% CI: 1.63-3.69) respectively Premature delivery time was associated with higher risk of MUO (OR = 3.11, 95% CI: 1.59-6.07), and delayed delivery time would increase the risk of MHO (OR = 3.51, 95% CI: 1.19-10.35) (**Figure 2** and **Table S3**). However, model 2 (**Figure 3** and **Table S4**) differently showed that those in the category of 0-5 months for breastfeeding duration were of higher risk of MHOO (OR = 1.27, 95% CI: 1.03-1.56) and lower risk of MUOO (OR = 0.78, 95% CI: 0.63-0.96). Sensitivity analysis showed similar results, with some associations turning out to have no significance (**Table S5** and **Table S6**).

The interaction analysis showed that girls in the high birth weight group had a higher risk of MUOO and MUO compared with boys, which was similarly found in MUO from premature delivery group and MHO from delayed delivery group (*p_interaction_* < 0.05).

#### 3.3.4 Lifestyle indicators

Multivariate analysis in model 1 (**Figure 2** and **Table S3**) and model 2 (**Figure 3** and **Table S4**) indicated that sleep time and screen time were associated with obesity phenotypes. Less than 9 hours of sleep duration per day was associated with a higher risk of MUOO both in boys (OR = 1.28, 95% CI: 1.06-1.55) and girls (OR = 1.29, 95% CI: 1.03-1.63). However, the association of insufficient sleep time with MUOO and MUO was found only in boys in the sensitivity analysis (**Table S5** and **Table S6**). In addition, more than 2 hours of screen time per day was observed to be associated with a higher risk of MHOO (OR = 1.25, 95% CI: 1.06-1.47) and MHO (OR = 1.32, 95% CI: 1.03-1.69) in boys, and higher prevalence of MHOO (OR = 1.33, 95% CI: 1.06-1.67) and MUO (OR = 1.47, 95% CI: 1.06-2.04) in girls, with which sensitivity analysis was similar (**Table S5** and **Table S6**).

The interaction analysis showed screen time had significant interaction with sex in the prevalence of MUOO and MUO (*p_interaction_* < 0.05).

## 4 Discussion

Given that not all individuals with the same degree of obesity had equivalent health risks, understanding how MHO and MUO were distributed and identifying those subgroups could not only be beneficial to providing specific intervention approaches but also to optimizing limited resources’ allocation, especially in China, which has the heaviest obesity burden. To our knowledge, this was the first study to investigate the prevalence of obesity phenotypes and their risk factors by international standards in a representative national sample of children and adolescents in China, and additionally explored sex differences. Our results showed that, in the general population, the prevalence of MHO, MUO, MHOO, and MUOO were 3.5%, 5.6%, 11.1%, and 13.0%, respectively, with higher risks in boys. Our study also confirmed an obviously higher prevalence of MHO at younger ages, and single-child status, parental unhealthy status, early life adverse experiences, and unhealthy lifestyles (insufficient sleep time and excessive screen time) were risk factors for MHO and MUO among children and adolescents as well. Moreover, boys were at higher risk of MUO when they were single children, while girls were more sensitive to MUO with risk factors like parental unhealthy status, adverse experiences in early life, and excessive screen time.

Demographic characteristics were considered as significant predictors of obesity phenotypes in the present study, especially in age, sex, and single-child status. First of all, our results showed that boys and single-child status were risk factors for MHO and MUO, which was consistent with previous studies (39). Moreover, boys with single-child status were indicated to be at higher risk of MUOO and MUO, implying that families would have better resource allocation, more coddling, and higher tolerance of bad behaviors for this group (39). The association between age and obesity phenotypes is worth exploring, with higher prevalence of MHO and MHOO in both 7-9 and 10-12 years compared with 13-18 years, while different results showed in MUO and MUOO among 7-9 years children. Previous studies have shown that younger children had a higher prevalence of being overweight and obese (40), while older adolescents were more likely to develop metabolic abnormalities (41, 42), meaning metabolic abnormalities and obesity had opposite trends in age. Additionally, the current definition of obesity phenotypes was mainly customized for the high-risk group of 10-18 years old (16), while most metabolic index values might be smaller at younger ages, thus less likely to reach cut-offs of abnormalities, possibly causing lower risk of MUO and MUOO at the 7-9 years group.

Obesity is a complex disease influenced by genetics and environment, where parents play an important role in both aspects. In our study, children and adolescents with overweight parents were found to be nearly twice as likely to develop MHO and MUO as those with non-overweight parents, and the conclusion was similar to previous evidence including cross-sectional and longitudinal studies (43, 44). On the one hand, parental obesity could predict similar obesity susceptibility in their children due to genetic background (45). On the other hand, obesity-related lifestyle and behaviors in parents could be easily passed to future generations through family socialization processes (46). Similar mechanisms and causes can also be applied to the interpretation of parental history of hypertension and diabetes, with behaviors of smoking and drinking on obesity phenotypes of their offspring. Furthermore, many countries tend to carry out randomized controlled trials of family-based interventions to reverse obesity among children and adolescents at present, and show great effectiveness, further confirming the importance of families and parents for children’s weight management (47). In addition, a greater effect of parental smoking on the risk of developing MUOO in girls was observed in our analysis, suggesting that girls were possibly more vulnerable to family environment and parental behaviors, which was also proved in some other studies (48).

Considering that the first 1000 days of life are the most critical period in determining the nutritional and health status of children throughout their lives, and are also closely related to BMI and metabolic status later in life (31, 49–54), we took birth status into account, as well as breastfeeding duration, showing caesarean and high birth weight as significant predictors of obesity phenotypes, with similar associations being weaker for breastfeeding duration and delivery time. For caesarean, published research suggested that it could increase the risk of later obesity and metabolic diseases just as our study has shown, due to inadequate transfer of the maternal microbiome to caesarean-born infants resulting in disturbed colonization with bacterial microflora within the skin and digestive tract (51, 52). As for high birth weight, there is related evidence that high birth weight infants have important changes in early development of adipose tissue metabolism and insulin sensitivity, leading to more accumulation of both subcutaneous and visceral fat, as well as insulin resistance, associated with later obesity and metabolic disorders (49, 50). In addition, breastfeeding duration may affect the occurrence of obesity phenotypes because it is directly related to hormone transmission related to lipid metabolism in breast milk (54). However, it was worth noting that our results showed insufficient breastfeeding time with the opposite effects on MHOO and MUOO. This might be attributed to the fact that we took at least 6 months of breast-feeding as the reference group based on recommendations of WHO (22), including participants who had been breastfed for a long time, while some previous studies indicated being breastfed for more than 12 months would possibly increase the risk of MHO and MUO (31). As for delivery time, there are relatively few studies on the association of obesity phenotypes, but it could be speculated that it is related to birth weight so affecting later health (53). More notably, our study found out early life status had a stronger impact on the MUO of girls, implying that more attention and intervention should be paid to the later health of girls who experienced early life adverse events.

Lifestyle change is a fundamental reason for the sharp increase in overweight and obesity in the world, and it is recognized as an intervenable factor in promoting health (21, 26). Our study identified insufficient sleep duration and excessive screen time as risk factors for MHO and MUO among children and adolescents. Among them, sleep time was closely related to obesity and metabolic status in the research evidence (26, 55), and it was confirmed in our study. Possible reasons include that short sleep duration would lead to changes in body hormone levels (leptin, insulin, ghrelin, cortisol) related to obesity and metabolism, accompanied by increased sedentary time, excessive food intake, and other unhealthy lifestyles additionally (13, 55). Plus, previous studies have also demonstrated that excessive screen time could increase the risk of MHO and MUO (25, 56, 57), as well as our study. Further, this study showed sex differences in the effect of screen time on the MUO, with girls at higher risk than boys. A study in Spain came to the same conclusion, arguing that girls were more sensitive to personal performance and social acceptance, and they were more willing to chat online to maintain social relationships, with alleviating symptoms of depression or anxiety by ingesting fast food (58). Unfortunately, this study did not observe the associations between other lifestyles and obesity phenotypes among children and adolescents.

Although the disease burden of MHO is lower in the long term, it is only a transient status, and would develop into MUO in most cases, emphasizing the significance to prevent and control the occurrence of both MHO and MUO (8). However, current studies on obesity phenotypes among children and adolescents are mostly cross-sectional studies, lacking longitudinal and interventional studies to confirm causality and explore effective interventions (11). Importantly, there is no internationally recognized complete standards on the definition of obesity phenotypes, and it is necessary to further optimize and promote the current definition standard (15). Furthermore, our study found significant sex differences in the prevalence of obesity phenotypes and their risk factors, implicating that targeting young people’s health from a sex perspective has considerable potential to reduce obesity phenotype problems, especially for later healthy intervenable lifestyle.

Despite its many merits, including a big sample, broader age span of children and adolescents, and diverse factors from family and individual level, there were also some limitations in our study. Firstly, cross-sectional studies can not demonstrate causality between obesity phenotypes and risk factors, which was inherent and unchangeable for the study design. Secondly, most of the information in this study depended on the questionnaire surveys, meaning recall bias existed inevitably, but we have used objective measures to judge the obesity phenotypes, reducing information bias of the key variables as far as possible. Thirdly, according to the recommended definition of obesity phenotypes, nutritional status and blood pressure have provided identified sex-specific cutoffs, while HDL-C, FPG, and TG failed to have sex-specific cutoffs, which may be a new direction for future research. Besides, while exploring the genetic and environmental factors of parents, children with adoptive parents are special groups that need to be considered, but we failed to collect data about it. However, the percentage of adopted children in China is very low (59), so this would not interfere with the main results. In addition, we only collected data on lifestyles in the last week, which might not well represent the long-term status. Additionally, we excluded some samples missing key information, but only less than 5% of the samples were abandoned, and we performed a test on differences of basic demographic information between the primary sample and final sample, showing no significance. Finally, although we have controlled for many covariates, there were still some other residual confounding factors unmeasured.

## 5 Conclusion

In conclusion, the prevalence of MHO, MUO, MHOO, and MUOO were 3.5%, 5.6%, 11.1%, and 13.0% respectively among 7–18-year-old children and adolescents in China, with higher risks in boys. Younger ages, single-child status, parental unhealthy status, early life adverse experiences, and unhealthy lifestyles (insufficient sleep time and excessive screen time) were considered as important risk factors of MHO and MUO. Moreover, significant sex differences in the prevalence of obesity phenotypes and their risk factors implicated it might be necessary to manage obesity phenotype problems among children and adolescents from a sex perspective.

## Data availability statement

The raw data supporting the conclusions of this article will be made available by the authors, without undue reservation.

## Ethics statement

The studies involving human participants were reviewed and approved by Ethical Committee of the Peking University (NO.IRB0000105213034). Written informed consent to participate in this study was provided by the participants’ legal guardian/next of kin.

## Author contributions

Conceptualization, YS and SC. Data curation, JD, PZ, NM, YL, and DS. Formal analysis, SC and JD. Funding acquisition, YS. Investigation, ZZ, YD, and JM. Methodology, SC and JD. Project administration, ZZ, YD, YS, and JM. Resources, ZZ, YD, YS, and JM. Supervision, YS and JM. Writing—original draft, SC. Writing—review and editing, JD, PZ, NM, YL, DS, ZZ, YD, JM, and YS. All authors contributed to the article and approved the submitted version.

## Funding

The present research was supported by the Natural Science Foundation of Beijing (Grant No. 7222247 to YS) and the Capital’s Funds for Health Improvement and Research (2022-1G-4251 to YS).

## Conflict of interest

The authors declare that the research was conducted in the absence of any commercial or financial relationships that could be construed as a potential conflict of interest.

## Publisher’s note

All claims expressed in this article are solely those of the authors and do not necessarily represent those of their affiliated organizations, or those of the publisher, the editors and the reviewers. Any product that may be evaluated in this article, or claim that may be made by its manufacturer, is not guaranteed or endorsed by the publisher.
