# Corrigendum: Sex differences in metabolically healthy and metabolically unhealthy obesity among Chinese children and adolescents

**DOI:** 10.3389/fendo.2022.1074024

**Published:** 2022-11-23

**Authors:** Shan Cai, Jiajia Dang, Panliang Zhong, Ning Ma, Yunfei Liu, Di Shi, Zhiyong Zou, Yanhui Dong, Jun Ma, Yi Song

**Affiliations:** ^1^ Institute of Child and Adolescent Health, School of Public Health, Peking University, Beijing, China; ^2^ National Health Commission Key Laboratory of Reproductive Health, Peking University, Beijing, China

**Keywords:** obesity phenotypes, metabolically healthy obesity, metabolically unhealthy obesity, children and adolescents, sex differences

In the published article, there was an error in **Table 1** as published. Some text in **Table 1** was marked in red, which was unnecessary. In addition, the explanation of the red text in the notes of **Table 1** was redundant (“Red text means that the distribution of the four nutritional status is different between boys and girls”). The corrected **Table 1** and its caption appear below.

**Table 1:** Characteristics of the study participants.

	Boys (N=7,750)	Girls (N=7,364)	Total (N=15,114)	*P-value*
**Demographic indicators**				
Age (years)				0.003
7-9	2702 (34.9)	2578 (35.0)	5280 (34.9)	
10-12	2101 (27.1)	1829 (24.8)	3930 (26.0)	
13-18	2947 (38.0)	2957 (40.2)	5904 (39.1)	
Residence				0.217
Rural	2930 (37.8)	2856 (38.8)	5786 (38.3)	
Urban	4820 (62.2)	4508 (61.2)	9328 (61.7)	
Single-child status				<0.001
Yes	5825 (75.2)	4942 (67.1)	10767 (71.2)	
No	1925 (24.8)	2422 (32.9)	4347 (28.8)	
Parental education level				<0.001
Senior high school and above	3903 (50.4)	3974 (54.0)	7877 (52.1)	
Junior high school and below	3847 (49.6)	3390 (46.0)	7237 (47.9)	
**Parental indicators**				
Parental Smoking				0.004
Yes	3234 (41.7)	3242 (44.0)	6476 (42.8)	
No	4516 (58.3)	4122 (56.0)	8638 (57.2)	
Parental drinking				0.824
Excessive	182 (2.3)	177 (2.4)	359 (2.4)	
Moderate	7568 (97.7)	7187 (97.6)	14755 (97.6)	
Parental overweight				0.035
Yes	2763 (35.7)	2747 (37.3)	5510 (36.5)	
No	4987 (64.3)	4617 (62.7)	9604 (63.5)	
Parental hypertension				0.008
Yes	428 (5.5)	482 (6.5)	910 (6.0)	
No	7322 (94.5)	6882 (93.5)	14204 (94.0)	
Parental diabetes				0.102
Yes	131 (1.7)	151 (2.1)	282 (1.9)	
No	7619 (98.3)	7213 (97.9)	14832 (98.1)	
**Early life indicators**				
Delivery model				<0.001
Caesarean	4175 (53.9)	3662 (49.7)	7837 (51.9)	
Eutocia	3575 (46.1)	3702 (50.3)	7277 (48.1)	
Delivery time				0.842
Premature delivery	143 (1.8)	140 (1.9)	283 (1.9)	
Delayed delivery	114 (1.5)	116 (1.6)	230 (1.5)	
Normal	7493 (96.7)	7108 (96.5)	14601 (96.6)	
Birthweight				<0.001
HBW	675 (8.7)	430 (5.8)	1105 (7.3)	
LBW	229 (3.0)	269 (3.7)	498 (3.3)	
NBW	6846 (88.3)	6665 (90.5)	13511 (89.4)	
Breastfeeding				0.239
0-5m	1721 (22.2)	1577 (21.4)	3298 (21.8)	
≥6m	6029 (77.8)	5787 (78.6)	11816 (78.2)	
**Lifestyle indicators**				
Fruits				0.048
<150g/d	5690 (73.4)	5301 (72.0)	10991 (72.7)	
≥150g/d	2060 (26.6)	2063 (28.0)	4123 (27.3)	
Vegetables				0.701
<300g/d	6357 (82.0)	6058 (82.3)	12415 (82.1)	
≥300g/d	1393 (18.0)	1306 (17.7)	2699 (17.9)	
Beverage				<0.001
>250ml/w	4695 (60.6)	3628 (49.3)	8323 (55.1)	
≤250ml/w	3055 (39.4)	3736 (50.7)	6791 (44.9)	
Sleep time				0.426
<9h/d	6370 (82.2)	6089 (82.7)	12459 (82.4)	
≥9h/d	1380 (17.8)	1275 (17.3)	2655 (17.6)	
Screen time				<0.001
>2h/d	1816 (23.4)	1333 (18.1)	3149 (20.8)	
≤2h/d	5934 (76.6)	6031 (81.9)	11965 (79.2)	
PA time				<0.001
<1h/d	5254 (67.8)	5643 (76.6)	10897 (72.1)	
≥1h/d	2496 (32.2)	1721 (23.4)	4217 (27.9)	
**Nutritional status**				<0.001
Obesity	1021 (13.2)	350 (4.8)	1371 (9.1)	
Overweight	1294 (16.7)	962 (13.1)	2256 (14.9)	
Normal weight	5157 (66.5)	5813 (78.9)	10970 (72.6)	
Thinness	278 (3.6)	239 (3.2)	517 (3.4)	
**Metabolic indicators**				
BP				0.633
Normal	6074 (78.4)	5795 (78.7)	11869 (78.5)	
Abnormal	1676 (21.6)	1569 (21.3)	3245 (21.5)	
FPG				<0.001
Normal	7556 (97.5)	7286 (98.9)	14842 (98.2)	
Abnormal	194 (2.5)	78 (1.1)	272 (1.8)	
TG				0.001
Normal	6573 (84.8)	6102 (82.9)	12675 (83.9)	
Abnormal	1177 (15.2)	1262 (17.1)	2439 (16.1)	
HDL-C				<0.001
Normal	6694 (86.4)	6562 (89.1)	13256 (87.7)	
Abnormal	1056 (13.6)	802 (10.9)	1858 (12.3)	
Metabolic status				0.113
Metabolically healthy	4673 (60.3)	4533 (61.6)	9206 (60.9)	
Metabolically unhealthy	3077 (39.7)	2831 (38.4)	5908 (39.1)	
**Nutritional and metabolic status**				<0.001
MHO	408 (5.3)	119 (1.6)	527 (3.5)	
MUO	613 (7.9)	231 (3.1)	844 (5.6)	
MHOW	699 (9.0)	446 (6.1)	1145 (7.6)	
MUOW	595 (7.7)	516 (7.0)	1111 (7.4)	
MHNW	3360 (43.4)	3786 (51.4)	7146 (47.3)	
MUNW	1797 (23.3)	2027 (27.5)	3824 (25.3)	
Thinness	278 (3.6)	239 (3.3)	517 (3.5)	

P-value was regarding comparison between boys and girls; NBW, normal birth weight; LBW, low birth weight; LBW, high birth weight; PA, physical activity; BP, blood pressure; FPG, fasting plasma glucose; TG, triglyceride; HDL-C, high density lipoprotein-cholesterol; MHO, metabolically healthy obesity; MUO, metabolically unhealthy obesity; MHOW, metabolically healthy overweight; MUOW, metabolically unhealthy overweight; MHNW, metabolically healthy normal weight; MUNW, metabolically unhealthy normal weight.

The authors apologize for this error and state that this does not change the scientific conclusions of the article in any way. The original article has been updated.

## Publisher’s note

All claims expressed in this article are solely those of the authors and do not necessarily represent those of their affiliated organizations, or those of the publisher, the editors and the reviewers. Any product that may be evaluated in this article, or claim that may be made by its manufacturer, is not guaranteed or endorsed by the publisher.

