# Supplementary material for: Sex differences in metabolically healthy and metabolically unhealthy obesity among Chinese children and adolescents
Source: Front Endocrinol (Lausanne). 2022 Oct 14;13:980332. doi: 10.3389/fendo.2022.980332 (PMC9613922; doi:10.3389/fendo.2022.980332)
Supplement: Supplementary file 1 [file DataSheet_1.docx]

**Supplementary**

Table S1. Association between MHO/MUO prevalence and risk factors by sex in univariate analysis

|  | Boys (N=7,750) | | Girls (N=7,364) | |
| --- | --- | --- | --- | --- |
|  | MHO | MUO | MHO | MUO |
| ***Demographic indicators*** |  |  |  |  |
| Age (years) |  |  |  |  |
| 7-9 | **2.97 (1.89,4.66)** | 1.08 (0.80,1.46) | **6.04 (2.77,13.16)** | 1.04 (0.66,1.64) |
| 10-12 | **2.02 (1.32,3.11)** | **1.50 (1.16,1.93)** | **3.44 (1.57,7.53)** | 1.45 (0.97,2.16) |
| 13-18 | 1.00 (ref) | 1.00 (ref) | 1.00 (ref) | 1.00 (ref) |
| Residence |  |  |  |  |
| Rural | 1.05 (0.51,2.17) | 1.44 (0.97,2.15) | 0.98 (0.43,2.27) | **1.82 (1.11,2.97)** |
| Urban | 1.00 (ref) | 1.00 (ref) | 1.00 (ref) | 1.00 (ref) |
| Single-child status |  |  |  |  |
| Yes | 1.11 (0.86,1.42) | **1.42 (1.14,1.76)** | **1.92 (1.18,3.10)** | 0.98 (0.73,1.31) |
| No | 1.00 (ref) | 1.00 (ref) | 1.00 (ref) | 1.00 (ref) |
| Parental education level |  |  |  |  |
| Senior high school and above | 1.08 (0.84,1.37) | 1.07 (0.89,1.29) | **1.64 (1.04,2.59)** | 1.09 (0.81,1.46) |
| Junior high school and below | 1.00 (ref) | 1.00 (ref) | 1.00 (ref) | 1.00 (ref) |
| ***Parental indicators*** |  |  |  |  |
| Parental Smoking |  |  |  |  |
| Yes | 1.10 (0.89,1.36) | 1.18 (0.99,1.40) | 0.94 (0.65,1.37) | **1.41 (1.08,1.85)** |
| No | 1.00 (ref) | 1.00 (ref) | 1.00 (ref) | 1.00 (ref) |
| Parental drinking |  |  |  |  |
| Excessive | 1.70 (0.99,2.90) | 0.63 (0.33,1.22) | 1.49 (0.63,3.50) | 0.59 (0.21,1.60) |
| Moderate | 1.00 (ref) | 1.00 (ref) | 1.00 (ref) | 1.00 (ref) |
| Parental overweight |  |  |  |  |
| Yes | **1.93 (1.57,2.39)** | **2.21 (1.86,2.63)** | **2.08 (1.42,3.06)** | **2.68 (2.03,3.55)** |
| No | 1.00 (ref) | 1.00 (ref) | 1.00 (ref) | 1.00 (ref) |
| Parental hypertension |  |  |  |  |
| Yes | 1.47 (0.97,2.21) | **1.62 (1.19,2.20)** | 1.47 (0.77,2.81) | 1.16 (0.71,1.89) |
| No | 1.00 (ref) | 1.00 (ref) | 1.00 (ref) | 1.00 (ref) |
| Parental diabetes |  |  |  |  |
| Yes | **2.06 (1.08,3.93)** | 1.60 (0.93,2.77) | 1.54 (0.55,4.32) | **2.67 (1.44,4.94)** |
| No | 1.00 (ref) | 1.00 (ref) | 1.00 (ref) | 1.00 (ref) |
| ***Early life indicators*** |  |  |  |  |
| Delivery model |  |  |  |  |
| Caesarean | **1.49 (1.19,1.87)** | **1.25 (1.05,1.50)** | **1.76 (1.17,2.65)** | **1.42 (1.08,1.87)** |
| Eutocia | 1.00 (ref) | 1.00 (ref) | 1.00 (ref) | 1.00 (ref) |
| Delivery time |  |  |  |  |
| Premature delivery | 1.22 (0.64,2.32) | 1.19 (0.67,2.10) | 0.42 (0.06,3.06) | **3.19 (1.72,5.94)** |
| Delayed delivery | 0.28 (0.07,1.16) | 1.13 (0.58,2.19) | **2.88 (1.01,8.20)** | 1.78 (0.76,4.14) |
| Normal | 1.00 (ref) | 1.00 (ref) | 1.00 (ref) | 1.00 (ref) |
| Birthweight |  |  |  |  |
| HBW | **2.09 (1.56,2.78)** | **1.36 (1.03,1.80)** | **2.64 (1.53,4.54)** | **2.72 (1.83,4.04)** |
| LBW | 0.78 (0.39,1.55) | 1.10 (0.68,1.78) | 0.80 (0.25,2.58) | 1.57 (0.84,2.95) |
| NBW | 1.00 (ref) | 1.00 (ref) | 1.00 (ref) | 1.00 (ref) |
| Breastfeeding |  |  |  |  |
| 0-5m | 1.05 (0.82,1.33) | 0.87 (0.70,1.07) | **1.55 (1.04,2.33)** | 0.81 (0.57,1.15) |
| ≥6m | 1.00 (ref) | 1.00 (ref) | 1.00 (ref) | 1.00 (ref) |
| ***Lifestyle indicators*** |  |  |  |  |
| Fruits |  |  |  |  |
| <150g/d | 1.04 (0.82,1.31) | 0.87 (0.73,1.05) | 0.91 (0.62,1.35) | 0.80 (0.60,1.06) |
| ≥150g/d | 1.00 (ref) | 1.00 (ref) | 1.00 (ref) | 1.00 (ref) |
| Vegetables |  |  |  |  |
| <300g/d | 0.88 (0.68,1.14) | 0.89 (0.72,1.20) | 0.83 (0.53,1.31) | 0.77 (0.56,1.06) |
| ≥300g/d | 1.00 (ref) | 1.00 (ref) | 1.00 (ref) | 1.00 (ref) |
| Beverage |  |  |  |  |
| >250ml/w | 1.16 (0.94,1.44) | 0.99 (0.83,1.18) | 0.89 (0.61,1.30) | 0.92 (0.70,1.20) |
| ≤250ml/w | 1.00 (ref) | 1.00 (ref) | 1.00 (ref) | 1.00 (ref) |
| Sleep time |  |  |  |  |
| <9h/d | 1.16 (0.90,1.50) | 1.14 (0.90,1.43) | 1.08 (0.70,1.68) | 1.00 (0.70,1.44) |
| ≥9h/d | 1.00 (ref) | 1.00 (ref) | 1.00 (ref) | 1.00 (ref) |
| Screen time |  |  |  |  |
| >2h/d | **1.33 (1.04,1.70)** | 1.01 (0.82,1.25) | 1.33 (0.84,2.10) | **1.48 (1.08,2.04)** |
| ≤2h/d | 1.00 (ref) | 1.00 (ref) | 1.00 (ref) | 1.00 (ref) |
| PA time |  |  |  |  |
| <1h/d | 0.92 (0.73,1.16) | **0.82 (0.68,0.97)** | 1.16 (0.75,1.81) | 0.99 (0.72,1.35) |
| ≥1h/d | 1.00 (ref) | 1.00 (ref) | 1.00 (ref) | 1.00 (ref) |

Notes: Obesity phenotypes include metabolically healthy obesity (MHO), and metabolically unhealthy obesity (MUO), taking the participants without MHO or MUO as the reference group. PA, physical activity; NBW, normal birth weight; LBW, low birth weight; LBW, high birth weight.

Table S2. Association between MHOO/MUOO prevalence and risk factors by sex in univariate analysis

|  | Boys (N=7,750) | | Girls (N=7,364) | |
| --- | --- | --- | --- | --- |
|  | MHOO | MUOO | MHOO | MUOO |
| ***Demographic indicators*** |  |  |  |  |
| Age (years) |  |  |  |  |
| 7-9 | **2.59 (1.94,3.45)** | **0.74 (0.58,0.94)** | **2.02 (1.42,2.87)** | 0.86 (0.65,1.15) |
| 10-12 | **2.22 (1.73,2.85)** | **1.26 (1.03,1.53)** | **1.65 (1.21,2.24)** | 1.27 (0.99,1.61) |
| 13-18 | 1.00 (ref) | 1.00 (ref) | 1.00 (ref) | 1.00 (ref) |
| Residence |  |  |  |  |
| Rural | 1.23 (0.68,2.25) | 1.24 (0.87,1.77) | 1.22 (0.72,2.05) | **1.55 (1.06,2.27)** |
| Urban | 1.00 (ref) | 1.00 (ref) | 1.00 (ref) | 1.00 (ref) |
| Single-child status |  |  |  |  |
| Yes | 1.15 (0.98,1.36) | **1.37 (1.17,1.61)** | 1.16 (0.95,1.43) | 0.94 (0.79,1.12) |
| No | 1.00 (ref) | 1.00 (ref) | 1.00 (ref) | 1.00 (ref) |
| Parental education level |  |  |  |  |
| Senior high school and above | **1.29 (1.10,1.51)** | **1.19 (1.03,1.37)** | **1.31 (1.07,1.62)** | 1.18 (0.99,1.41) |
| Junior high school and below | 1.00 (ref) | 1.00 (ref) | 1.00 (ref) | 1.00 (ref) |
| ***Parental indicators*** |  |  |  |  |
| Parental Smoking |  |  |  |  |
| Yes | 1.03 (0.90,1.19) | 1.08 (0.95,1.23) | **1.23 (1.03,1.47)** | **1.34 (1.14,1.56)** |
| No | 1.00 (ref) | 1.00 (ref) | 1.00 (ref) | 1.00 (ref) |
| Parental drinking |  |  |  |  |
| Excessive | 1.40 (0.94,2.08) | 0.87 (0.56,1.35) | 1.22 (0.74,2.02) | 1.18 (0.74,1.88) |
| Moderate | 1.00 (ref) | 1.00 (ref) | 1.00 (ref) | 1.00 (ref) |
| Parental overweight |  |  |  |  |
| Yes | **1.91 (1.66,2.19)** | **2.16 (1.89,2.46)** | **2.19 (1.83,2.63)** | **2.03 (1.73,2.39)** |
| No | 1.00 (ref) | 1.00 (ref) | 1.00 (ref) | 1.00 (ref) |
| Parental hypertension |  |  |  |  |
| Yes | 1.11 (0.83,1.50) | **1.59 (1.24,2.04)** | **1.59 (1.16,2.17)** | **1.41 (1.06,1.87)** |
| No | 1.00 (ref) | 1.00 (ref) | 1.00 (ref) | 1.00 (ref) |
| Parental diabetes |  |  |  |  |
| Yes | **1.77 (1.11,2.82)** | 1.44 (0.91,2.28) | **1.95 (1.19,3.19)** | **1.69 (1.06,2.69)** |
| No | 1.00 (ref) | 1.00 (ref) | 1.00 (ref) | 1.00 (ref) |
| ***Early life indicators*** |  |  |  |  |
| Delivery model |  |  |  |  |
| Caesarean | **1.19 (1.03,1.37)** | 1.10 (0.96,1.26) | **1.31 (1.09,1.58)** | 1.11 (0.94,1.30) |
| Eutocia | 1.00 (ref) | 1.00 (ref) | 1.00 (ref) | 1.00 (ref) |
| Delivery time |  |  |  |  |
| Premature delivery | 1.00 (0.63,1.58) | 0.92 (0.57,1.49) | 0.78 (0.39,1.57) | **1.81 (1.12,2.91)** |
| Delayed delivery | 0.85 (0.48,1.52) | 1.04 (0.61,1.76) | 1.23 (0.61,2.48) | **1.94 (1.17,3.21)** |
| Normal | 1.00 (ref) | 1.00 (ref) | 1.00 (ref) | 1.00 (ref) |
| Birthweight |  |  |  |  |
| HBW | **1.60 (1.29,1.98)** | 1.12 (0.89,1.41) | **1.91 (1.40,2.60)** | **1.67 (1.25,2.23)** |
| LBW | 0.76 (0.49,1.17) | 1.20 (0.84,1.71) | 0.84 (0.50,1.40) | 0.80 (0.51,1.27) |
| NBW | 1.00 (ref) | 1.00 (ref) | 1.00 (ref) | 1.00 (ref) |
| Breastfeeding |  |  |  |  |
| 0-5m | 0.95 (0.81,1.12) | 0.89 (0.76,1.05) | **1.34 (1.10,1.64)** | 0.83 (0.67,1.02) |
| ≥6m | 1.00 (ref) | 1.00 (ref) | 1.00 (ref) | 1.00 (ref) |
| ***Lifestyle indicators*** |  |  |  |  |
| Fruits |  |  |  |  |
| <150g/d | 0.93 (0.80,1.08) | 0.94 (0.81,1.08) | 0.98 (0.81,1.19) | 0.94 (0.79,1.11) |
| ≥150g/d | 1.00 (ref) | 1.00 (ref) | 1.00 (ref) | 1.00 (ref) |
| Vegetables |  |  |  |  |
| <300g/d | 0.87 (0.73,1.03) | 0.87 (0.74,1.02) | 0.87 (0.70,1.08) | 0.98 (0.80,1.20) |
| ≥300g/d | 1.00 (ref) | 1.00 (ref) | 1.00 (ref) | 1.00 (ref) |
| Beverage |  |  |  |  |
| >250ml/w | 1.04 (0.91,1.20) | 1.13 (0.99,1.30) | 1.12 (0.94,1.34) | 0.98 (0.84,1.15) |
| ≤250ml/w | 1.00 (ref) | 1.00 (ref) | 1.00 (ref) | 1.00 (ref) |
| Sleep time |  |  |  |  |
| <9h/d | 1.05 (0.89,1.25) | **1.23 (1.02,1.48)** | 1.11 (0.89,1.40) | 1.23 (0.98,1.54) |
| ≥9h/d | 1.00 (ref) | 1.00 (ref) | 1.00 (ref) | 1.00 (ref) |
| Screen time |  |  |  |  |
| >2h/d | **1.25 (1.06,1.46)** | 0.97 (0.83,1.14) | **1.32 (1.06,1.65)** | 1.13 (0.93,1.39) |
| ≤2h/d | 1.00 (ref) | 1.00 (ref) | 1.00 (ref) | 1.00 (ref) |
| PA time |  |  |  |  |
| <1h/d | 0.91 (0.78,1.05) | **0.85 (0.74,0.97)** | 1.04 (0.84,1.29) | 0.99 (0.83,1.19) |
| ≥1h/d | 1.00 (ref) | 1.00 (ref) | 1.00 (ref) | 1.00 (ref) |

Notes: Obesity phenotypes include metabolically healthy overweight and obesity (MHOO), and metabolically unhealthy overweight and obesity (MUOO), taking the participants without MHOO or MUOO as the reference group. PA, physical activity; NBW, normal birth weight; LBW, low birth weight; LBW, high birth weight.

Table S3. Association between MHO/MUO prevalence and risk factors by sex in multivariate analysis

|  | Boys (N=7,750) | | Girls (N=7,364) | | *P-interaciton* | |
| --- | --- | --- | --- | --- | --- | --- |
|  | MHO | MUO | MHO | MUO | MHO | MUO |
| ***Demographic indicators*** |  |  |  |  |  |  |
| Age (years) |  |  |  |  |  |  |
| 7-9 | **3.40 (2.16,5.35)** | 1.20 (0.89,1.62) | **6.41 (2.94,13.95)** | 1.12 (0.72,1.75) | 0.374 | 0.763 |
| 10-12 | **2.22 (1.44,3.42)** | **1.62 (1.25,2.09)** | **3.53 (1.60,7.78)** | 1.46 (0.98,2.18) | 0.542 | 0.348 |
| 13-18 | 1.00 (ref) | 1.00 (ref) | 1.00 (ref) | 1.00 (ref) |  |  |
| Residence |  |  |  |  |  |  |
| Rural | 1.04 (0.60,1.81) | 1.35 (0.94,1.94) | 1.40 (0.71,2.76) | **1.76 (1.11,2.78)** | 0.404 | 0.053 |
| Urban | 1.00 (ref) | 1.00 (ref) | 1.00 (ref) | 1.00 (ref) |  |  |
| Single-child status |  |  |  |  |  |  |
| Yes | 1.18 (0.91,1.54) | **1.56 (1.24,1.95)** | **2.11 (1.27,3.49)** | 1.12 (0.82,1.54) | 0.093 | 0.017* |
| No | 1.00 (ref) | 1.00 (ref) | 1.00 (ref) | 1.00 (ref) |  |  |
| Parental education level |  |  |  |  |  |  |
| Senior high school and above | 0.97 (0.75,1.25) | 0.93 (0.76,1.14) | 1.32 (0.82,2.13) | 0.99 (0.72,1.35) | 0.461 | 0.559 |
| Junior high school and below | 1.00 (ref) | 1.00 (ref) | 1.00 (ref) | 1.00 (ref) |  |  |
| ***Parental indicators*** |  |  |  |  |  |  |
| Parental Smoking |  |  |  |  |  |  |
| Yes | 1.06 (0.85,1.32) | 1.16 (0.97,1.39) | 0.90 (0.61,1.31) | **1.34 (1.02,1.76)** | 0.742 | 0.172 |
| No | 1.00 (ref) | 1.00 (ref) | 1.00 (ref) | 1.00 (ref) |  |  |
| Parental drinking |  |  |  |  |  |  |
| Excessive | 1.55 (0.89,2.70) | 0.60 (0.31,1.16) | 1.48 (0.61,3.58) | 0.50 (0.18,1.38) | 0.966 | 0.938 |
| Moderate | 1.00 (ref) | 1.00 (ref) | 1.00 (ref) | 1.00 (ref) |  |  |
| Parental overweight |  |  |  |  |  |  |
| Yes | **1.93 (1.55,2.40)** | **2.28 (1.90,2.73)** | **2.04 (1.38,3.03)** | **2.51 (1.88,3.35)** | 0.480 | 0.260 |
| No | 1.00 (ref) | 1.00 (ref) | 1.00 (ref) | 1.00 (ref) |  |  |
| Parental hypertension |  |  |  |  |  |  |
| Yes | 1.24 (0.81,1.91) | 1.37 (0.99,1.90) | 1.30 (0.65,2.58) | 0.89 (0.53,1.48) | 0.946 | 0.395 |
| No | 1.00 (ref) | 1.00 (ref) | 1.00 (ref) | 1.00 (ref) |  |  |
| Parental diabetes |  |  |  |  |  |  |
| Yes | 1.84 (0.94,3.59) | 1.29 (0.73,2.28) | 1.33 (0.45,3.93) | **2.51 (1.31,4.80)** | 0.740 | 0.190 |
| No | 1.00 (ref) | 1.00 (ref) | 1.00 (ref) | 1.00 (ref) |  |  |
| ***Early life indicators*** |  |  |  |  |  |  |
| Delivery model |  |  |  |  |  |  |
| Caesarean | **1.51 (1.20,1.90)** | **1.31 (1.09,1.57)** | **1.63 (1.08,2.46)** | **1.53 (1.15,2.03)** | 0.682 | 0.854 |
| Eutocia | 1.00 (ref) | 1.00 (ref) | 1.00 (ref) | 1.00 (ref) |  |  |
| Delivery time |  |  |  |  |  |  |
| Premature delivery | 1.50 (0.77,2.94) | 1.22 (0.67,2.22) | 0.47 (0.06,3.61) | **3.11 (1.59,6.07)** | 0.300 | 0.038* |
| Delayed delivery | **0.23 (0.05,0.96)** | 0.99 (0.50,1.95) | **3.51 (1.19,10.35)** | 1.48 (0.62,3.52) | 0.007* | 0.268 |
| Normal | 1.00 (ref) | 1.00 (ref) | 1.00 (ref) | 1.00 (ref) |  |  |
| Birthweight |  |  |  |  |  |  |
| HBW | **1.99 (1.48,2.67)** | 1.28 (0.96,1.70) | **2.39 (1.36,4.18)** | **2.45 (1.63,3.69)** | 0.541 | 0.003* |
| LBW | 0.73 (0.36,1.50) | 1.13 (0.69,1.86) | 0.87 (0.26,2.88) | 1.26 (0.64,2.47) | 0.936 | 0.400 |
| NBW | 1.00 (ref) | 1.00 (ref) | 1.00 (ref) | 1.00 (ref) |  |  |
| Breastfeeding |  |  |  |  |  |  |
| 0-5m | 0.95 (0.74,1.21) | 0.82 (0.66,1.02) | 1.43 (0.94,2.18) | 0.73 (0.51,1.05) | 0.222 | 0.475 |
| ≥6m | 1.00 (ref) | 1.00 (ref) | 1.00 (ref) | 1.00 (ref) |  |  |
| ***Lifestyle indicators*** |  |  |  |  |  |  |
| Fruits |  |  |  |  |  |  |
| <150g/d | 1.07 (0.84,1.37) | 0.92 (0.76,1.12) | 0.90 (0.59,1.37) | 0.81 (0.60,1.09) | 0.376 | 0.457 |
| ≥150g/d | 1.00 (ref) | 1.00 (ref) | 1.00 (ref) | 1.00 (ref) |  |  |
| Vegetables |  |  |  |  |  |  |
| <300g/d | 0.84 (0.64,1.11) | 0.96 (0.77,1.21) | 0.78 (0.48,1.26) | 0.84 (0.60,1.18) | 0.668 | 0.518 |
| ≥300g/d | 1.00 (ref) | 1.00 (ref) | 1.00 (ref) | 1.00 (ref) |  |  |
| Beverage |  |  |  |  |  |  |
| >250ml/w | 1.16 (0.93,1.44) | 0.98 (0.82,1.18) | 0.91 (0.62,1.34) | 0.96 (0.73,1.27) | 0.286 | 0.895 |
| ≤250ml/w | 1.00 (ref) | 1.00 (ref) | 1.00 (ref) | 1.00 (ref) |  |  |
| Sleep time |  |  |  |  |  |  |
| <9h/d | 1.24 (0.96,1.61) | 1.24 (0.98,1.57) | 1.25 (0.80,1.96) | 1.06 (0.73,1.52) | 0.803 | 0.413 |
| ≥9h/d | 1.00 (ref) | 1.00 (ref) | 1.00 (ref) | 1.00 (ref) |  |  |
| Screen time |  |  |  |  |  |  |
| >2h/d | **1.32 (1.03,1.69)** | 0.97 (0.79,1.20) | 1.44 (0.91,2.30) | **1.47 (1.06,2.04)** | 0.754 | 0.017* |
| ≤2h/d | 1.00 (ref) | 1.00 (ref) | 1.00 (ref) | 1.00 (ref) |  |  |
| PA time |  |  |  |  |  |  |
| <1h/d | 0.92 (0.72,1.16) | 0.84 (0.69,1.01) | 1.20 (0.76,1.88) | 1.13 (0.82,1.57) | 0.475 | 0.369 |
| ≥1h/d | 1.00 (ref) | 1.00 (ref) | 1.00 (ref) | 1.00 (ref) |  |  |

Notes: Obesity phenotypes include metabolically healthy obesity (MHO), and metabolically unhealthy obesity (MUO), taking the participants without MHO or MUO as the reference group. Adjusted for age, residence, single-child status, parental education level, parental smoking, parental drinking, parental overweight, parental hypertension, parental diabetes, delivery model, delivery time, birth weight, breastfeeding, fruits, vegetables, beverage, sleep time, screen time, physical activity (PA) time. *Significant interaction with sex in the whole model. NBW, normal birth weight; LBW, low birth weight; LBW, high birth weight.

Table S4. Association between MHOO/MUOO prevalence and risk factors by sex in multivariate analysis

|  | Boys (N=7,750) | | Girls (N=7,364) | | *P-interaciton* | |
| --- | --- | --- | --- | --- | --- | --- |
|  | MHOO | MUOO | MHOO | MUOO | MHOO | MUOO |
| ***Demographic indicators*** |  |  |  |  |  |  |
| Age (years) |  |  |  |  |  |  |
| 7-9 | **2.73 (2.04,3.65)** | 0.80 (0.63,1.03) | **2.06 (1.46,2.91)** | 0.94 (0.70,1.25) | 0.010* | 0.217 |
| 10-12 | **2.30 (1.78,2.95)** | **1.34 (1.10,1.64)** | **1.65 (1.21,2.24)** | **1.34 (1.06,1.70)** | 0.018* | 0.811 |
| 13-18 | 1.00 (ref) | 1.00 (ref) | 1.00 (ref) | 1.00 (ref) |  |  |
| Residence |  |  |  |  |  |  |
| Rural | 1.28 (0.78,2.09) | 1.15 (0.82,1.63) | 1.26 (0.82,1.94) | 1.43 (0.99,2.05) | 0.806 | 0.052 |
| Urban | 1.00 (ref) | 1.00 (ref) | 1.00 (ref) | 1.00 (ref) |  |  |
| Single-child status |  |  |  |  |  |  |
| Yes | **1.21 (1.02,1.44)** | **1.49 (1.26,1.76)** | 1.22 (0.99,1.51) | 1.03 (0.86,1.24) | 0.314 | <0.001* |
| No | 1.00 (ref) | 1.00 (ref) | 1.00 (ref) | 1.00 (ref) |  |  |
| Parental education level |  |  |  |  |  |  |
| Senior high school and above | **1.18 (1.01,1.40)** | 1.07 (0.92,1.24) | 1.12 (0.90,1.40) | 1.09 (0.91,1.31) | 0.120 | 0.310 |
| Junior high school and below | 1.00 (ref) | 1.00 (ref) | 1.00 (ref) | 1.00 (ref) |  |  |
| ***Parental indicators*** |  |  |  |  |  |  |
| Parental Smoking |  |  |  |  |  |  |
| Yes | 0.98 (0.85,1.13) | 1.05 (0.92,1.21) | 1.19 (0.99,1.43) | **1.28 (1.09,1.50)** | 0.072 | 0.017* |
| No | 1.00 (ref) | 1.00 (ref) | 1.00 (ref) | 1.00 (ref) |  |  |
| Parental drinking |  |  |  |  |  |  |
| Excessive | 1.34 (0.89,2.01) | 0.83 (0.53,1.31) | 1.08 (0.65,1.80) | 1.06 (0.66,1.71) | 0.826 | 0.280 |
| Moderate | 1.00 (ref) | 1.00 (ref) | 1.00 (ref) | 1.00 (ref) |  |  |
| Parental overweight |  |  |  |  |  |  |
| Yes | **1.89 (1.64,2.19)** | **2.22 (1.93,2.56)** | **2.09 (1.73,2.52)** | **1.94 (1.65,2.29)** | 0.139 | 0.732 |
| No | 1.00 (ref) | 1.00 (ref) | 1.00 (ref) | 1.00 (ref) |  |  |
| Parental hypertension |  |  |  |  |  |  |
| Yes | 0.97 (0.71,1.32) | **1.33 (1.02,1.73)** | 1.31 (0.94,1.82) | 1.18 (0.88,1.58) | 0.144 | 0.716 |
| No | 1.00 (ref) | 1.00 (ref) | 1.00 (ref) | 1.00 (ref) |  |  |
| Parental diabetes |  |  |  |  |  |  |
| Yes | **1.70 (1.05,2.76)** | 1.15 (0.71,1.86) | 1.64 (0.98,2.73) | 1.41 (0.87,2.29) | 0.656 | 0.598 |
| No | 1.00 (ref) | 1.00 (ref) | 1.00 (ref) | 1.00 (ref) |  |  |
| ***Early life indicators*** |  |  |  |  |  |  |
| Delivery model |  |  |  |  |  |  |
| Caesarean | **1.23 (1.06,1.42)** | **1.15 (1.01,1.33)** | **1.34 (1.10,1.62)** | **1.20 (1.01,1.42)** | 0.802 | 0.474 |
| Eutocia | 1.00 (ref) | 1.00 (ref) | 1.00 (ref) | 1.00 (ref) |  |  |
| Delivery time |  |  |  |  |  |  |
| Premature delivery | 1.11 (0.69,1.79) | 0.89 (0.53,1.49) | 0.78 (0.38,1.61) | **1.96 (1.19,3.23)** | 0.407 | 0.088 |
| Delayed delivery | 0.73 (0.41,1.30) | 0.97 (0.57,1.66) | 1.18 (0.57,2.43) | **1.74 (1.04,2.90)** | 0.278 | 0.064 |
| Normal | 1.00 (ref) | 1.00 (ref) | 1.00 (ref) | 1.00 (ref) |  |  |
| Birthweight |  |  |  |  |  |  |
| HBW | **1.51 (1.21,1.88)** | 1.05 (0.83,1.34) | **1.70 (1.24,2.33)** | **1.50 (1.12,2.01)** | 0.404 | 0.019* |
| LBW | 0.80 (0.51,1.25) | 1.30 (0.89,1.90) | 0.85 (0.50,1.44) | 0.70 (0.43,1.12) | 0.973 | 0.191 |
| NBW | 1.00 (ref) | 1.00 (ref) | 1.00 (ref) | 1.00 (ref) |  |  |
| Breastfeeding |  |  |  |  |  |  |
| 0-5m | 0.87 (0.74,1.03) | **0.84 (0.71,0.99)** | **1.27 (1.03,1.56)** | **0.78 (0.63,0.96)** | 0.077 | 0.366 |
| ≥6m | 1.00 (ref) | 1.00 (ref) | 1.00 (ref) | 1.00 (ref) |  |  |
| ***Lifestyle indicators*** |  |  |  |  |  |  |
| Fruits |  |  |  |  |  |  |
| <150g/d | 0.99 (0.84,1.16) | 1.00 (0.86,1.17) | 1.00 (0.81,1.22) | 0.94 (0.79,1.13) | 0.928 | 0.695 |
| ≥150g/d | 1.00 (ref) | 1.00 (ref) | 1.00 (ref) | 1.00 (ref) |  |  |
| Vegetables |  |  |  |  |  |  |
| <300g/d | 0.88 (0.74,1.06) | 0.91 (0.76,1.08) | 0.84 (0.67,1.07) | 1.03 (0.83,1.27) | 0.828 | 0.357 |
| ≥300g/d | 1.00 (ref) | 1.00 (ref) | 1.00 (ref) | 1.00 (ref) |  |  |
| Beverage |  |  |  |  |  |  |
| >250ml/w | 1.07 (0.93,1.24) | 1.12 (0.97,1.29) | 1.12 (0.93,1.35) | 1.00 (0.85,1.18) | 0.319 | 0.140 |
| ≤250ml/w | 1.00 (ref) | 1.00 (ref) | 1.00 (ref) | 1.00 (ref) |  |  |
| Sleep time |  |  |  |  |  |  |
| <9h/d | 1.15 (0.97,1.37) | **1.28 (1.06,1.55)** | 1.19 (0.94,1.50) | **1.29 (1.03,1.63)** | 0.384 | 0.540 |
| ≥9h/d | 1.00 (ref) | 1.00 (ref) | 1.00 (ref) | 1.00 (ref) |  |  |
| Screen time |  |  |  |  |  |  |
| >2h/d | **1.25 (1.06,1.47)** | 0.93 (0.79,1.09) | **1.33 (1.06,1.67)** | 1.12 (0.91,1.38) | 0.225 | 0.041* |
| ≤2h/d | 1.00 (ref) | 1.00 (ref) | 1.00 (ref) | 1.00 (ref) |  |  |
| PA time |  |  |  |  |  |  |
| <1h/d | 0.93 (0.80,1.09) | 0.87 (0.75,1.01) | 1.08 (0.87,1.35) | 1.05 (0.87,1.28) | 0.337 | 0.265 |
| ≥1h/d | 1.00 (ref) | 1.00 (ref) | 1.00 (ref) | 1.00 (ref) |  |  |

Obesity phenotypes include metabolically healthy overweight and obesity (MHOO), and metabolically unhealthy overweight and obesity (MUOO), taking the participants without MHOO or MUOO as the reference group. Adjusted for age, residence, single-child status, parental education level, parental smoking, parental drinking, parental overweight, parental hypertension, parental diabetes, delivery model, delivery time, birth weight, breastfeeding, fruits, vegetables, beverage, sleep time, screen time, physical activity (PA) time. *Significant interaction with sex in the whole model. NBW, normal birth weight; LBW, low birth weight; LBW, high birth weight.

Table S5. Association between MHO/MUO prevalence and risk factors by sex in sensitive analysis

|  | Boys (N=4,381) | | Girls (N=4,136) | |
| --- | --- | --- | --- | --- |
|  | MHO | MUO | MHO | MUO |
| ***Demographic indicators*** |  |  |  |  |
| Age (years) |  |  |  |  |
| 7-9 | **2.80 (1.81,4.33)** | 0.78 (0.54,1.14) | **5.91 (2.76,12.66)** | 0.75 (0.43,1.28) |
| 10-12 | **2.14 (1.40,3.27)** | 1.33 (0.97,1.82) | **4.01 (1.83,8.81)** | 1.40 (0.88,2.23) |
| 13-18 | 1.00 (ref) | 1.00 (ref) | 1.00 (ref) | 1.00 (ref) |
| Residence |  |  |  |  |
| Rural | 0.80 (0.50,1.28) | 0.86 (0.47,1.57) | 1.19 (0.63,2.26) | 1.42 (0.71,2.85) |
| Urban | 1.00 (ref) | 1.00 (ref) | 1.00 (ref) | 1.00 (ref) |
| Single-child status |  |  |  |  |
| Yes | 1.23 (0.94,1.62) | **1.59 (1.24,2.03)** | **2.26 (1.36,3.77)** | 1.21 (0.86,1.68) |
| No | 1.00 (ref) | 1.00 (ref) | 1.00 (ref) | 1.00 (ref) |
| Parental education level |  |  |  |  |
| Senior high school and above | 0.96 (0.74,1.25) | 0.87 (0.69,1.08) | 1.20 (0.74,1.95) | 0.89 (0.64,1.25) |
| Junior high school and below | 1.00 (ref) | 1.00 (ref) | 1.00 (ref) | 1.00 (ref) |
| ***Parental indicators*** |  |  |  |  |
| Parental Smoking |  |  |  |  |
| Yes | 1.02 (0.82,1.28) | 1.08 (0.89,1.31) | 0.96 (0.65,1.41) | **1.40 (1.05,1.87)** |
| No | 1.00 (ref) | 1.00 (ref) | 1.00 (ref) | 1.00 (ref) |
| Parental drinking |  |  |  |  |
| Excessive | 1.72 (0.94,3.14) | 0.63 (0.31,1.29) | 1.64 (0.66,4.09) | 0.47 (0.16,1.38) |
| Moderate | 1.00 (ref) | 1.00 (ref) | 1.00 (ref) | 1.00 (ref) |
| Parental overweight |  |  |  |  |
| Yes | **2.30 (1.83,2.88)** | **2.57 (2.11,3.13)** | **2.30 (1.55,3.42)** | **2.62 (1.95,3.53)** |
| No | 1.00 (ref) | 1.00 (ref) | 1.00 (ref) | 1.00 (ref) |
| Parental hypertension |  |  |  |  |
| Yes | 1.24 (0.79,1.95) | 1.40 (0.97,2.02) | 1.38 (0.68,2.78) | 1.02 (0.59,1.74) |
| No | 1.00 (ref) | 1.00 (ref) | 1.00 (ref) | 1.00 (ref) |
| Parental diabetes |  |  |  |  |
| Yes | **2.07 (1.01,4.27)** | 1.31 (0.68,2.52) | 1.41 (0.46,4.30) | **2.64 (1.29,5.39)** |
| No | 1.00 (ref) | 1.00 (ref) | 1.00 (ref) | 1.00 (ref) |
| ***Early life indicators*** |  |  |  |  |
| Delivery model |  |  |  |  |
| Caesarean | **1.48 (1.17,1.89)** | **1.24 (1.02,1.52)** | **1.73 (1.14,2.62)** | **1.62 (1.20,2.18)** |
| Eutocia | 1.00 (ref) | 1.00 (ref) | 1.00 (ref) | 1.00 (ref) |
| Delivery time |  |  |  |  |
| Premature delivery | 1.54 (0.77,3.11) | 1.25 (0.64,2.45) | 0.45 (0.06,3.55) | **2.89 (1.38,6.04)** |
| Delayed delivery | **0.23 (0.06,0.97)** | 0.82 (0.39,1.72) | **3.64 (1.18,11.21)** | 1.81 (0.71,4.62) |
| Normal | 1.00 (ref) | 1.00 (ref) | 1.00 (ref) | 1.00 (ref) |
| Birthweight |  |  |  |  |
| HBW | **2.01 (1.47,2.75)** | 1.30 (0.95,1.78) | **2.15 (1.21,3.84)** | **2.24 (1.45,3.46)** |
| LBW | 0.77 (0.37,1.62) | 1.22 (0.70,2.13) | 0.76 (0.22,2.55) | 1.00 (0.48,2.11) |
| NBW | 1.00 (ref) | 1.00 (ref) | 1.00 (ref) | 1.00 (ref) |
| Breastfeeding |  |  |  |  |
| 0-5m | 0.91 (0.71,1.18) | **0.78 (0.62,0.99)** | 1.38 (0.90,2.11) | 0.70 (0.48,1.02) |
| ≥6m | 1.00 (ref) | 1.00 (ref) | 1.00 (ref) | 1.00 (ref) |
| ***Lifestyle indicators*** |  |  |  |  |
| Fruits |  |  |  |  |
| <150g/d | 1.06 (0.82,1.36) | 1.00 (0.80,1.23) | 0.86 (0.56,1.32) | 0.84 (0.61,1.15) |
| ≥150g/d | 1.00 (ref) | 1.00 (ref) | 1.00 (ref) | 1.00 (ref) |
| Vegetables |  |  |  |  |
| <300g/d | 0.86 (0.65,1.15) | 0.93 (0.72,1.18) | 0.84 (0.51,1.36) | 0.91 (0.64,1.31) |
| ≥300g/d | 1.00 (ref) | 1.00 (ref) | 1.00 (ref) | 1.00 (ref) |
| Beverage |  |  |  |  |
| >250ml/w | 1.20 (0.96,1.51) | 1.04 (0.85,1.26) | 0.92 (0.62,1.37) | 0.94 (0.70,1.26) |
| ≤250ml/w | 1.00 (ref) | 1.00 (ref) | 1.00 (ref) | 1.00 (ref) |
| Sleep time |  |  |  |  |
| <9h/d | 1.29 (0.98,1.68) | **1.31 (1.01,1.69)** | 1.32 (0.84,2.07) | 1.06 (0.72,1.56) |
| ≥9h/d | 1.00 (ref) | 1.00 (ref) | 1.00 (ref) | 1.00 (ref) |
| Screen time |  |  |  |  |
| >2h/d | **1.32 (1.02,1.71)** | 1.02 (0.81,1.28) | 1.50 (0.93,2.42) | **1.49 (1.05,2.11)** |
| ≤2h/d | 1.00 (ref) | 1.00 (ref) | 1.00 (ref) | 1.00 (ref) |
| PA time |  |  |  |  |
| <1h/d | 0.99 (0.77,1.26) | 0.92 (0.75,1.13) | 1.16 (0.73,1.85) | 1.16 (0.82,1.62) |
| ≥1h/d | 1.00 (ref) | 1.00 (ref) | 1.00 (ref) | 1.00 (ref) |

Notes: Obesity phenotypes include metabolically healthy obesity (MHO), and metabolically unhealthy obesity (MUO), taking the participants who were metabolically healthy normal weight (MHNW) as the reference group. Adjusted for age, residence, single-child status, parental education level, parental smoking, parental drinking, parental overweight, parental hypertension, parental diabetes, delivery model, delivery time, birth weight, breastfeeding, fruits, vegetables, beverage, sleep time, screen time, physical activity (PA) time. NBW, normal birth weight; LBW, low birth weight; LBW, high birth weight.

Table S6. Association between MHOO/MUOO prevalence and risk factors by sex in sensitive analysis

|  | Boys (N=5,675) | | Girls (N=5,098) | |
| --- | --- | --- | --- | --- |
|  | MHOO | MUOO | MHOO | MUOO |
| ***Demographic indicators*** |  |  |  |  |
| Age (years) |  |  |  |  |
| 7-9 | **2.00 (1.55,2.59)** | **0.50 (0.37,0.68)** | **1.75 (1.30,2.34)** | **0.57 (0.40,0.82)** |
| 10-12 | **1.88 (1.48,2.38)** | 0.99 (0.77,1.27) | **1.71 (1.28,2.29)** | 1.14 (0.85,1.53) |
| 13-18 | 1.00 (ref) | 1.00 (ref) | 1.00 (ref) | 1.00 (ref) |
| Residence |  |  |  |  |
| Rural | 0.92 (0.69,1.23) | 0.66 (0.32,1.37) | 0.95 (0.70,1.28) | 0.91 (0.44,1.92) |
| Urban | 1.00 (ref) | 1.00 (ref) | 1.00 (ref) | 1.00 (ref) |
| Single-child status |  |  |  |  |
| Yes | **1.23 (1.03,1.47)** | **1.48 (1.23,1.79)** | **1.26 (1.02,1.56)** | 1.11 (0.91,1.36) |
| No | 1.00 (ref) | 1.00 (ref) | 1.00 (ref) | 1.00 (ref) |
| Parental education level |  |  |  |  |
| Senior high school and above | **1.19 (1.01,1.41)** | 1.06 (0.89,1.26) | 1.04 (0.83,1.29) | 1.06 (0.86,1.30) |
| Junior high school and below | 1.00 (ref) | 1.00 (ref) | 1.00 (ref) | 1.00 (ref) |
| ***Parental indicators*** |  |  |  |  |
| Parental Smoking |  |  |  |  |
| Yes | 0.96 (0.83,1.11) | 1.02 (0.87,1.18) | 1.20 (0.99,1.45) | **1.24 (1.04,1.48)** |
| No | 1.00 (ref) | 1.00 (ref) | 1.00 (ref) | 1.00 (ref) |
| Parental drinking |  |  |  |  |
| Excessive | 1.38 (0.90,2.12) | 0.92 (0.56,1.50) | 1.12 (0.66,1.89) | 1.09 (0.66,1.80) |
| Moderate | 1.00 (ref) | 1.00 (ref) | 1.00 (ref) | 1.00 (ref) |
| Parental overweight |  |  |  |  |
| Yes | **1.93 (1.66,2.24)** | **2.29 (1.96,2.68)** | **2.16 (1.79,2.61)** | **1.98 (1.66,2.37)** |
| No | 1.00 (ref) | 1.00 (ref) | 1.00 (ref) | 1.00 (ref) |
| Parental hypertension |  |  |  |  |
| Yes | 0.98 (0.71,1.36) | **1.35 (1.01,1.81)** | 1.29 (0.92,1.81) | 1.16 (0.84,1.59) |
| No | 1.00 (ref) | 1.00 (ref) | 1.00 (ref) | 1.00 (ref) |
| Parental diabetes |  |  |  |  |
| Yes | **1.69 (1.02,2.82)** | 1.1 (0.64,1.89) | 1.61 (0.95,2.73) | 1.46 (0.85,2.49) |
| No | 1.00 (ref) | 1.00 (ref) | 1.00 (ref) | 1.00 (ref) |
| ***Early life indicators*** |  |  |  |  |
| Delivery model |  |  |  |  |
| Caesarean | **1.21 (1.04,1.40)** | 1.09 (0.93,1.27) | **1.37 (1.13,1.66)** | **1.26 (1.05,1.51)** |
| Eutocia | 1.00 (ref) | 1.00 (ref) | 1.00 (ref) | 1.00 (ref) |
| Delivery time |  |  |  |  |
| Premature delivery | 1.16 (0.70,1.91) | 0.98 (0.56,1.72) | 0.76 (0.36,1.58) | **1.84 (1.06,3.19)** |
| Delayed delivery | 0.68 (0.38,1.24) | 0.81 (0.44,1.48) | 1.24 (0.58,2.61) | 1.76 (0.97,3.19) |
| Normal | 1.00 (ref) | 1.00 (ref) | 1.00 (ref) | 1.00 (ref) |
| Birthweight |  |  |  |  |
| HBW | **1.50 (1.20,1.89)** | 1.08 (0.83,1.40) | **1.51 (1.09,2.09)** | 1.24 (0.90,1.71) |
| LBW | 0.86 (0.54,1.37) | 1.38 (0.90,2.11) | 0.79 (0.46,1.35) | 0.64 (0.38,1.07) |
| NBW | 1.00 (ref) | 1.00 (ref) | 1.00 (ref) | 1.00 (ref) |
| Breastfeeding |  |  |  |  |
| 0-5m | 0.88 (0.75,1.04) | **0.82 (0.68,0.99)** | **1.24 (1.01,1.53)** | **0.76 (0.61,0.96)** |
| ≥6m | 1.00 (ref) | 1.00 (ref) | 1.00 (ref) | 1.00 (ref) |
| ***Lifestyle indicators*** |  |  |  |  |
| Fruits |  |  |  |  |
| <150g/d | 1.00 (0.85,1.18) | 1.07 (0.90,1.27) | 0.93 (0.76,1.15) | 0.91 (0.75,1.11) |
| ≥150g/d | 1.00 (ref) | 1.00 (ref) | 1.00 (ref) | 1.00 (ref) |
| Vegetables |  |  |  |  |
| <300g/d | 0.89 (0.74,1.08) | 0.89 (0.73,1.08) | 0.90 (0.71,1.14) | 1.15 (0.91,1.45) |
| ≥300g/d | 1.00 (ref) | 1.00 (ref) | 1.00 (ref) | 1.00 (ref) |
| Beverage |  |  |  |  |
| >250ml/w | 1.11 (0.96,1.29) | **1.17 (1.01,1.37)** | 1.11 (0.92,1.33) | 0.99 (0.83,1.18) |
| ≤250ml/w | 1.00 (ref) | 1.00 (ref) | 1.00 (ref) | 1.00 (ref) |
| Sleep time |  |  |  |  |
| <9h/d | 1.16 (0.97,1.39) | **1.31 (1.07,1.61)** | 1.21 (0.96,1.53) | 1.28 (0.99,1.64) |
| ≥9h/d | 1.00 (ref) | 1.00 (ref) | 1.00 (ref) | 1.00 (ref) |
| Screen time |  |  |  |  |
| >2h/d | **1.25 (1.06,1.48)** | 0.97 (0.81,1.16) | **1.36 (1.08,1.71)** | 1.17 (0.93,1.46) |
| ≤2h/d | 1.00 (ref) | 1.00 (ref) | 1.00 (ref) | 1.00 (ref) |
| PA time |  |  |  |  |
| <1h/d | 1.01 (0.86,1.18) | 0.95 (0.81,1.11) | 1.07 (0.86,1.33) | 1.06 (0.86,1.31) |
| ≥1h/d | 1.00 (ref) | 1.00 (ref) | 1.00 (ref) | 1.00 (ref) |

Notes: Obesity phenotypes include metabolically healthy overweight and obesity (MHOO), and metabolically unhealthy overweight and obesity (MUOO), taking the participants who were metabolically healthy normal weight (MHNW) as the reference group. Adjusted for age, residence, single-child status, parental education level, parental smoking, parental drinking, parental overweight, parental hypertension, parental diabetes, delivery model, delivery time, birth weight, breastfeeding, fruits, vegetables, beverage, sleep time, screen time, physical activity (PA) time. NBW, normal birth weight; LBW, low birth weight; LBW, high birth weight.

**Table S7. Different** **metabolic abnormal status in girls**

|  | **Metabolic indicators** | | | |
| --- | --- | --- | --- | --- |
|  | **BP** | **FPG** | **TG** | **HDL-C** |
| ***Demographic indicators*** |  |  |  |  |
| Age (years) |  |  |  |  |
| 7-9 | **0.38 (0.29,0.50)** | 0.89 (0.66,1.19) | **0.46 (0.31,0.68)** | **0.65 (0.47,0.90)** |
| 10-12 | **0.77 (0.63,0.95)** | 0.97 (0.70,1.33) | 0.85 (0.63,1.14) | 0.91 (0.72,1.16) |
| 13-18 | 1.00 (ref) | 1.00 (ref) | 1.00 (ref) | 1.00 (ref) |
| Residence |  |  |  |  |
| Rural | **0.39 (0.21,0.71)** | 0.89 (0.69,1.14) | **4.78 (1.32,17.3**) | 1.15 (0.57,2.33) |
| Urban | 1.00 (ref) | 1.00 (ref) | 1.00 (ref) | 1.00 (ref) |
| Single-child status |  |  |  |  |
| Yes | 1.10 (0.96,1.27) | 1.01 (0.77,1.32) | 1.06 (0.86,1.30) | 1.05 (0.88,1.25) |
| No | 1.00 (ref) | 1.00 (ref) | 1.00 (ref) | 1.00 (ref) |
| Parental education level |  |  |  |  |
| Senior high school and above | 1.02 (0.88,1.17) | 1.07 (0.83,1.37) | 1.09 (0.89,1.35) | 0.92 (0.77,1.10) |
| Junior high school and below | 1.00 (ref) | 1.00 (ref) | 1.00 (ref) | 1.00 (ref) |
| ***Parental indicators*** |  |  |  |  |
| Parental Smoking |  |  |  |  |
| Yes | 1.05 (0.93,1.19) | 1.02 (0.79,1.31) | 1.13 (0.94,1.37) | 1.07 (0.91,1.26) |
| No | 1.00 (ref) | 1.00 (ref) | 1.00 (ref) | 1.00 (ref) |
| Parental drinking |  |  |  |  |
| Excessive | 0.88 (0.60,1.30) | 0.83 (0.34,2.02) | 0.89 (0.48,1.64) | 0.98 (0.56,1.70) |
| Moderate | 1.00 (ref) | 1.00 (ref) | 1.00 (ref) | 1.00 (ref) |
| Parental overweight |  |  |  |  |
| Yes | 1.10 (0.97,1.25) | 1.05 (0.81,1.36) | 1.12 (0.93,1.36) | **1.41 (1.19,1.67)** |
| No | 1.00 (ref) | 1.00 (ref) | 1.00 (ref) | 1.00 (ref) |
| Parental hypertension |  |  |  |  |
| Yes | 1.00 (0.79,1.26) | 1.03 (0.63,1.70) | 1.31 (0.93,1.85) | 1.22 (0.88,1.68) |
| No | 1.00 (ref) | 1.00 (ref) | 1.00 (ref) | 1.00 (ref) |
| Parental diabetes |  |  |  |  |
| Yes | 1.30 (0.87,1.93) | 1.43 (0.67,3.05) | 1.51 (0.85,2.69) | 1.02 (0.57,1.82) |
| No | 1.00 (ref) | 1.00 (ref) | 1.00 (ref) | 1.00 (ref) |
| ***Early life indicators*** |  |  |  |  |
| Delivery model |  |  |  |  |
| Caesarean | 1.00 (0.88,1.13) | 0.91 (0.71,1.18) | 1.05 (0.87,1.27) | 1.03 (0.87,1.21) |
| Eutocia | 1.00 (ref) | 1.00 (ref) | 1.00 (ref) | 1.00 (ref) |
| Delivery time |  |  |  |  |
| Premature delivery | 1.21 (0.77,1.90) | 1.20 (0.51,2.81) | 1.43 (0.78,2.60) | 1.63 (0.98,2.72) |
| Delayed delivery | 1.26 (0.81,1.97) | 1.29 (0.52,3.17) | 1.61 (0.87,3.00) | 1.46 (0.83,2.57) |
| Normal | 1.00 (ref) | 1.00 (ref) | 1.00 (ref) | 1.00 (ref) |
| Birthweight |  |  |  |  |
| HBW | 0.90 (0.69,1.16) | 0.86 (0.49,1.52) | 0.92 (0.61,1.38) | 0.91 (0.63,1.31) |
| LBW | 0.96 (0.69,1.33) | 0.94 (0.47,1.86) | 0.87 (0.54,1.42) | 1.33 (0.89,1.99) |
| NBW | 1.00 (ref) | 1.00 (ref) | 1.00 (ref) | 1.00 (ref) |
| Breastfeeding |  |  |  |  |
| 0-5m | 0.87 (0.74,1.02) | 1.05 (0.77,1.41) | 0.99 (0.78,1.25) | 0.93 (0.76,1.14) |
| ≥6m | 1.00 (ref) | 1.00 (ref) | 1.00 (ref) | 1.00 (ref) |
| ***Lifestyle indicators*** |  |  |  |  |
| Fruits |  |  |  |  |
| <150g/d | 0.94 (0.82,1.08) | 1.00 (0.76,1.32) | 0.82 (0.67,1.01) | 0.95 (0.79,1.13) |
| ≥150g/d | 1.00 (ref) | 1.00 (ref) | 1.00 (ref) | 1.00 (ref) |
| Vegetables |  |  |  |  |
| <300g/d | 1.11 (0.94,1.30) | 1.01 (0.73,1.41) | 1.07 (0.84,1.36) | 0.98 (0.80,1.20) |
| ≥300g/d | 1.00 (ref) | 1.00 (ref) | 1.00 (ref) | 1.00 (ref) |
| Beverage |  |  |  |  |
| >250ml/w | 0.96 (0.85,1.09) | 1.00 (0.78,1.28) | 0.98 (0.81,1.18) | 0.99 (0.84,1.16) |
| ≤250ml/w | 1.00 (ref) | 1.00 (ref) | 1.00 (ref) | 1.00 (ref) |
| Sleep time |  |  |  |  |
| <9h/d | **1.25 (1.04,1.49)** | 1.09 (0.78,1.54) | **0.76 (0.59,0.99)** | 0.92 (0.73,1.16) |
| ≥9h/d | 1.00 (ref) | 1.00 (ref) | 1.00 (ref) | 1.00 (ref) |
| Screen time |  |  |  |  |
| >2h/d | 1.07 (0.91,1.25) | 0.92 (0.66,1.29) | 1.23 (0.97,1.54) | 0.97 (0.79,1.20) |
| ≤2h/d | 1.00 (ref) | 1.00 (ref) | 1.00 (ref) | 1.00 (ref) |
| PA time |  |  |  |  |
| <1h/d | 0.99 (0.85,1.14) | 0.93 (0.69,1.24) | 1.05 (0.85,1.31) | 1.04 (0.86,1.25) |
| ≥1h/d | 1.00 (ref) | 1.00 (ref) | 1.00 (ref) | 1.00 (ref) |

Notes: Univariate analysis with normal metabolic group as reference group; BP, blood pressure; FPG, fasting plasma glucose; TG, triglyceride; HDL-C, high density lipoprotein-cholesterol.

**Table S8. Different metabolic abnormal status in boys**

|  | **Metabolic indicators** | | | |
| --- | --- | --- | --- | --- |
|  | **BP** | **FPG** | **TG** | **HDL-C** |
| ***Demographic indicators*** |  |  |  |  |
| Age (years) |  |  |  |  |
| 7-9 | **0.36 (0.28,0.46)** | 0.88 (0.48,1.61) | **0.56 (0.38,0.83)** | **0.41 (0.30,0.55)** |
| 10-12 | **0.70 (0.57,0.86)** | 1.51 (0.93,2.45) | 0.99 (0.74,1.32) | **0.49 (0.39,0.62)** |
| 13-18 | 1.00 (ref) | 1.00 (ref) | 1.00 (ref) | 1.00 (ref) |
| Residence |  |  |  |  |
| Rural | 0.59 (0.32,1.09) | 0.48 (0.22,1.05) | **31.28 (9.01,108.53)** | 0.95 (0.42,2.14) |
| Urban | 1.00 (ref) | 1.00 (ref) | 1.00 (ref) | 1.00 (ref) |
| Single-child status |  |  |  |  |
| Yes | 1.20 (1.04,1.38) | 0.96 (0.68,1.37) | 1.22 (0.97,1.52) | 1.12 (0.94,1.33) |
| No | 1.00 (ref) | 1.00 (ref) | 1.00 (ref) | 1.00 (ref) |
| Parental education level |  |  |  |  |
| Senior high school and above | 1.02 (0.89,1.17) | 0.96 (0.69,1.34) | 1.16 (0.94,1.43) | 0.86 (0.73,1.01) |
| Junior high school and below | 1.00 (ref) | 1.00 (ref) | 1.00 (ref) | 1.00 (ref) |
| ***Parental indicators*** |  |  |  |  |
| Parental Smoking |  |  |  |  |
| Yes | 1.01 (0.90,1.14) | 1.05 (0.78,1.41) | 1.08 (0.89,1.31) | 0.97 (0.84,1.13) |
| No | 1.00 (ref) | 1.00 (ref) | 1.00 (ref) | 1.00 (ref) |
| Parental drinking |  |  |  |  |
| Excessive | 1.13 (0.79,1.62) | 0.51 (0.12,2.10) | 1.16 (0.64,2.13) | 0.84 (0.50,1.41) |
| Moderate | 1.00 (ref) | 1.00 (ref) | 1.00 (ref) | 1.00 (ref) |
| Parental overweight |  |  |  |  |
| Yes | **1.38 (1.22,1.56)** | 1.02 (0.75,1.39) | **1.53 (1.25,1.86)** | **1.25 (1.07,1.46)** |
| No | 1.00 (ref) | 1.00 (ref) | 1.00 (ref) | 1.00 (ref) |
| Parental hypertension |  |  |  |  |
| Yes | **1.68 (1.33,2.11)** | 1.04 (0.60,1.80) | 0.81 (0.53,1.24) | 1.11 (0.82,1.49) |
| No | 1.00 (ref) | 1.00 (ref) | 1.00 (ref) | 1.00 (ref) |
| Parental diabetes |  |  |  |  |
| Yes | 1.06 (0.68,1.65) | 1.09 (0.38,3.10) | 1.06 (0.53,2.12) | 0.75 (0.41,1.35) |
| No | 1.00 (ref) | 1.00 (ref) | 1.00 (ref) | 1.00 (ref) |
| ***Early life indicators*** |  |  |  |  |
| Delivery model |  |  |  |  |
| Caesarean | 1.10 (0.97,1.24) | 0.95 (0.70,1.28) | 0.90 (0.74,1.10) | 0.95 (0.82,1.11) |
| Eutocia | 1.00 (ref) | 1.00 (ref) | 1.00 (ref) | 1.00 (ref) |
| Delivery time |  |  |  |  |
| Premature delivery | 1.05 (0.67,1.64) | 1.51 (0.59,3.86) | 1.40 (0.73,2.68) | 1.18 (0.71,1.96) |
| Delayed delivery | 1.36 (0.86,2.14) | 0.61 (0.15,2.58) | 1.22 (0.58,2.59) | 0.88 (0.47,1.66) |
| Normal | 1.00 (ref) | 1.00 (ref) | 1.00 (ref) | 1.00 (ref) |
| Birthweight |  |  |  |  |
| HBW | 1.04 (0.85,1.28) | 0.89 (0.51,1.55) | 0.98 (0.69,1.39) | 0.86 (0.65,1.14) |
| LBW | 1.30 (0.94,1.80) | 1.40 (0.63,3.12) | 1.12 (0.65,1.92) | 1.13 (0.73,1.74) |
| NBW | 1.00 (ref) | 1.00 (ref) | 1.00 (ref) | 1.00 (ref) |
| Breastfeeding |  |  |  |  |
| 0-5m | 0.93 (0.80,1.09) | 0.87 (0.60,1.26) | 0.85 (0.67,1.07) | 0.90 (0.75,1.09) |
| ≥6m | 1.00 (ref) | 1.00 (ref) | 1.00 (ref) | 1.00 (ref) |
| ***Lifestyle indicators*** |  |  |  |  |
| Fruits |  |  |  |  |
| <150g/d | 1.13 (0.99,1.29) | 1.04 (0.74,1.46) | 1.14 (0.92,1.40) | 0.88 (0.75,1.03) |
| ≥150g/d | 1.00 (ref) | 1.00 (ref) | 1.00 (ref) | 1.00 (ref) |
| Vegetables |  |  |  |  |
| <300g/d | 1.01 (0.87,1.18) | 1.33 (0.88,2.02) | 0.90 (0.71,1.13) | 0.91 (0.75,1.09) |
| ≥300g/d | 1.00 (ref) | 1.00 (ref) | 1.00 (ref) | 1.00 (ref) |
| Beverage |  |  |  |  |
| >250ml/w | **1.14 (1.01,1.29)** | 1.12 (0.81,1.55) | 1.09 (0.89,1.33) | 1.09 (0.93,1.27) |
| ≤250ml/w | 1.00 (ref) | 1.00 (ref) | 1.00 (ref) | 1.00 (ref) |
| Sleep time |  |  |  |  |
| <9h/d | **1.19 (1.01,1.41)** | 0.97 (0.62,1.52) | 1.07 (0.81,1.42) | 1.18 (0.94,1.48) |
| ≥9h/d | 1.00 (ref) | 1.00 (ref) | 1.00 (ref) | 1.00 (ref) |
| Screen time |  |  |  |  |
| >2h/d | 1.06 (0.92,1.22) | 1.06 (0.74,1.53) | 1.01 (0.80,1.27) | **0.81 (0.67,0.97)** |
| ≤2h/d | 1.00 (ref) | 1.00 (ref) | 1.00 (ref) | 1.00 (ref) |
| PA time |  |  |  |  |
| <1h/d | 1.09 (0.96,1.24) | 0.89 (0.66,1.22) | 0.91 (0.74,1.11) | 0.91 (0.79,1.06) |
| ≥1h/d | 1.00 (ref) | 1.00 (ref) | 1.00 (ref) | 1.00 (ref) |

Notes: Univariate analysis with normal metabolic group as reference group; BP, blood pressure; FPG, fasting plasma glucose; TG, triglyceride; HDL-C, high density lipoprotein-cholesterol.

**Table S9** **Different metabolic states in** **overweight versus obese groups**

| **Metabolic indicators** | **Overweight (N=2256)** | **Obesity (N=1371)** | ***P-value*** |
| --- | --- | --- | --- |
| BP |  |  | <0.001 |
| Normal | 1566 (69.4) | 774 (56.5) |  |
| Abnormal | 690 (30.6) | 597 (43.5) |  |
| FPG |  |  | 0.069 |
| Normal | 2210 (98.0) | 1330 (97.0) |  |
| Abnormal | 46 (2.0) | 41 (3.0) |  |
| TG |  |  | 0.001 |
| Normal | 1848 (81.9) | 1058 (77.2) |  |
| Abnormal | 408 (18.1) | 313 (22.8) |  |
| HDL-C |  |  | <0.001 |
| Normal | 1891 (83.8) | 1074 (78.3) |  |
| Abnormal | 365 (16.2) | 297 (21.7) |  |

Notes: *P-value* was regarding comparison between overweight versus obese groups; BP, blood pressure; FPG, fasting plasma glucose; TG, triglyceride; HDL-C, high density lipoprotein-cholesterol.
